# Identification of glycerol-3-phosphate dehydrogenase 1 as a tumour suppressor in human breast cancer

**DOI:** 10.18632/oncotarget.21087

**Published:** 2017-09-19

**Authors:** Cefan Zhou, Jing Yu, Ming Wang, Jing Yang, Hui Xiong, Huang Huang, Dongli Wu, Shimeng Hu, Yefu Wang, Xing-Zhen Chen, Jingfeng Tang

**Affiliations:** ^1^ Institute of Biomedical and Pharmaceutical Sciences, Key Laboratory of Fermentation Engineering (Ministry of Education), Hubei Provincial Cooperative Innovation Center of Industrial Fermentation, Hubei Key Laboratory of Industrial Microbiology, Hubei University of Technology, Wuhan, Hubei, China; ^2^ The State Key Laboratory of Virology, College of Life Sciences, Wuhan University, Wuhan, Hubei, China; ^3^ Department of Clinical Laboratory, Hubei Cancer Hospital, Wuhan, Hubei, China; ^4^ Department of Clinical Laboratory, Renmin Hospital of Wuhan University, Wuhan, Hubei, China; ^5^ Institute for Immunology, Tsinghua University, Beijing, China; ^6^ XiLi People's Hospital, Shenzhen, Guangdong, China; ^7^ Membrane Protein Disease Research Group, Department of Physiology, Faculty of Medicine and Dentistry, University of Alberta, Edmonton, AB, Canada

**Keywords:** prognostic significance, biomarker, cell proliferation, survival, meta-analysis

## Abstract

In the present study, we found the mRNA expression level of glycerol-3-phosphate dehydrogenase (GPD1) was significantly downregulated in human breast cancer patients. Patients with reduced GPD1 expression exhibited poorer overall metastatic relapse-free survival (*p* = 0.0013). Further Cox proportional hazard model analysis revealed that the reduced expression of GPD1 is an independent predictor of overall survival in oestrogen receptor-positive (*p* = 0.0027, HR = 0.91, 95% CI = 0.85–0.97, *N* = 3,917) and nodal-negative (*p* = 0.0013, HR = 0.87, 95% CI = 0.80–0.95, *N* = 2,456) breast cancer patients. We also demonstrated that GPD1 was a direct target of miR-370, which was significantly upregulated in human breast cancer. We further showed that exogenous expression of GPD1 in human MCF-7 and MDA-MB-231 breast cancer cells significantly inhibited cell proliferation, migration, and invasion. Our results, therefore, suggest a novel tumour suppressor function for GPD1 and contribute to the understanding of cancer metabolism.

## INTRODUCTION

GPD1 encodes cytoplasmic NAD-dependent glycerol-3-phosphate dehydrogenase 1, a 349-amino-acid 37.5 kD protein that catalyses the conversion of dihydroxyacetone phosphate (DHAP) derived from glucose to glycerol-3-phosphate and Nicotinamide adenine dinucleotide (NAD^+^), which is then acylated to form triglycerides [1]. Importantly, the products glycerol 3-phosphate and glycerol act as a backbone for lipid biosynthesis [2]. GPD1, together with a mitochondrial enzyme named GPD2, also has an important role in the transport of reducing equivalents from the cytosol to the mitochondria [3]. GPD1 is widely distributed in tissues, including various regions of the brain and internal tissues, with the highest levels in mesentery fat, subcutaneous fat, and the duodenum [4, 5]. GPD1-deficient mice have been reported to exhibit enhanced exercise capacity through increased lipid oxidation via activation of Adenosine 5‘-monophosphate (AMP) -activated protein kinase (AMPK) [6]. In addition, they exhibit decreased adiposity and body weight, despite a sufficient food supply [7]. Thus, GPD1 is considered a key element that connects carbohydrate and lipid metabolism. Abnormal GPD1 activity contributes to the increase or decrease of triacylglycerol (TAG) synthesis in obese patients [8] and plays a crucial role in hypertriglyceridemia, fatty liver, hepatic fibrosis, hepatomegaly and steatohepatitis [3, 4]. However, little is known about the role of GPD1 in human cancers, particularly in human breast cancer.

Breast cancer is a heterogeneous disease accompanied by differences in clinical, molecular and biological features, which creates a challenge for prognosis and treatment [9]. Estrogen receptor positive (ER+) subtype (Oestrogen receptor or hormone receptor subtype) is the most vital discriminator of breast cancer, accounting for nearly 75% of all breast cancer cases [10]. Although progress has been made in the diagnosis and treatment of breast cancer, the prognosis and survival for most patients, particularly those with metastases, have not dramatically improved [11, 12]. In addition, it is believed that during chemotherapy, drug resistance frequently develops and impairs the successful treatment of breast cancer. Therefore, there is an urgent need for the identification of diagnostic markers and clarification of the potential cellular and molecular mechanisms underlying tumour metastasis, as well as for the development of new therapeutic strategies for improving patient survival and overall quality of life.

In this study, we first investigated the expression profile of GPD1 in human breast cancer using the Cancer Genome Atlas (TCGA) database and the prognostic significance of GPD1 expression for the survival of human breast cancer patients through a meta-analysis of publicly available mRNA expression data. Then, GPD1expression was verified with real-time quantitative PCR (qRT-PCR), western blotting and immunohistochemistry. In addition, we confirmed that GPD1 can inhibit breast cancer cell proliferation, migration, and invasion. We also identified the relationship between GPD1 and miR-370. To the best of our knowledge, the data generated in this study represent the first report of a correlation between the presence of GPD1 and the survival of human breast cancer patients.

## RESULTS

### The expression pattern of GPD1 in human breast cancer

**Figure 1:**
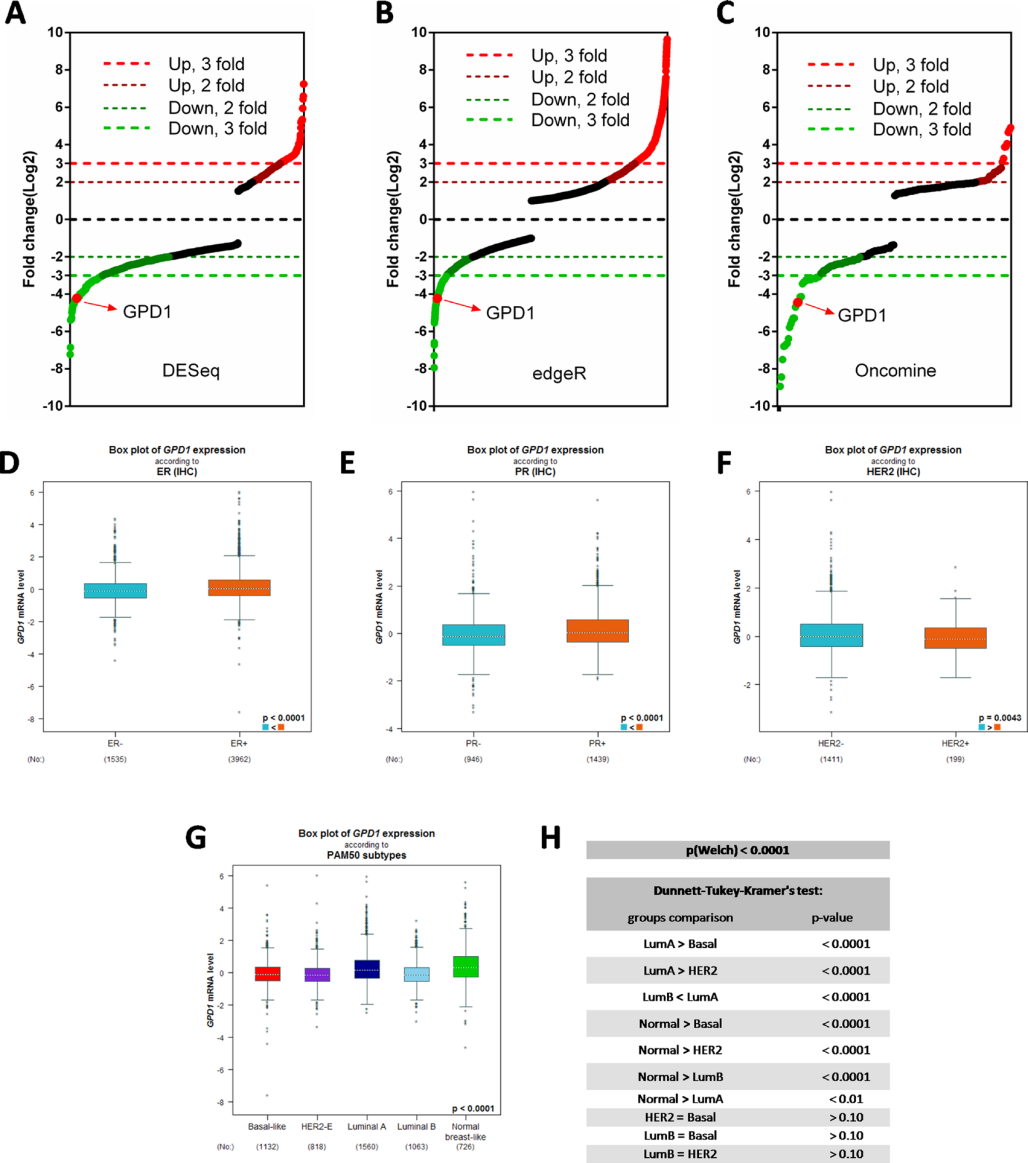
The expression pattern of GPD1 in human breast cancer (**A**–**B**) Distribution of fold changes illustrated in gene expression profiles for the 30 breast cancer cases (10 normal and 20 tumours). The log2 values were calculated for each sample by normalizing to read counts alone (log2Fold Change). Gene expression analysis was performed using R version 3.2.2 software with DESeq package (*p*-value < 0.01 and log2 Fold Change > 3) (A) and edgeR package (*p*-value < 0.01 and log2 Fold Change > 3) (B). RNAseq data were downloaded from TCGA database. (**C**) Distribution of fold changes for the top 200 significantly changed genes in human breast cancer obtained from a Oncomine dataset which contains 19,273 measured genes from 2,136 breast cancer samples based on Illumina HumanHT-12 V3.0 R2 Array. (**D**–**F**) Expression analysis for GPD1 with positive versus negative receptor IHC status (ER, PR and HER2) (*n* = 5,510). (**G**–**H**) Expression analysis for GPD1 according to PAM50 subtypes (*n* = 5,299). Dunnett-Tukey-Kramer's test was used for group comparisons.

We first queried the expression pattern of GPD1 in human breast cancer. Therefore, publicly available RNA-sequence datasets for 30 breast cancer and 10 normal breast tissue samples were downloaded fromTCGA database. These datasets were further used to generate a heatmap for further analysis with the R program (version 3.2.2) (Supplementary Figure 1). GPD1 expression was significantly downregulated in both “DESeq” and “edgeR” algorithms (Figure 1A and 1B, log2fold change > 3 and *p* < 0.01). Furthermore, a consistent result for GPD1 expression in breast cancer was found in the Oncomine database (Figure 1C and Supplementary Figure 1D).

Immunohistochemistry (IHC) markers together with clinicopathological indexes are used to classify breast cancer and predict disease outcome [13]. By using the Bc-GenExMiner v4.0 database [14], we analysed the GPD1 expression in human breast cancer patients with several splitting criteria, including receptor status and molecular subtype. The results showed that GPD1 expression was significantly higher in oestrogen receptor-positive (ER+, *p* < 0.0001, N = 5,497) (Figure 1D), progesterone receptor-positive (PR+, *p* < 0.0001, *N* = 2,385) (Figure 1E) and HER2 receptor-negative (HER2-, *p* = 0.0043, *N* = 1,610) (Figure 1F) samples. In addition, in the breast cancer subtypes according to the prediction analysis of microarray 50 (PAM50) [15], luminal A subtype showed the highest GPD1 mRNA level compared with luminal B, HER2-enriched and basal-like subtypes (Figure 1G–1H). We also analysed the expression of GPD1 in human breast cancer patients with different ages (*N* = 3,552), Scarff-Bloom-Richardson grading (SBR, *N* = 3,470) and Nottingham prognostic indexes (*N* = 1,762) (Supplementary Figure 2).

### GPD1 expression level is correlated with breast cancer patient overall survival

**Figure 2:**
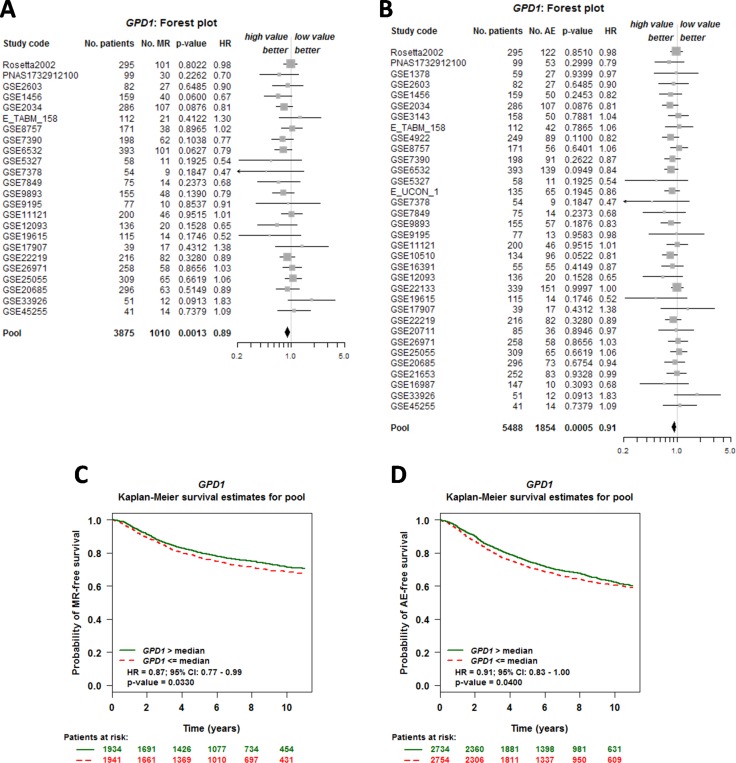
GPD1 expression is correlated with overall survival of breast cancer patients (**A**–**B**) Forest plot shows the impact of GPD1 expression on MR- (A) and AE-free (B) survival. (**C**–**D**) Kaplan-Meier survival curves for the association between GPD1 expression and the probability of MR- and AE- free survival. The green curve represents the 50% of patients with higher GPD1 expression than the median expression level. In contrast, the red dashed curve represents the 50% of patients with lower GPD1 expression than the median expression level. “Patients at risk” refers to patients who are at risk of the event occurrence, such as death or metastatic relapse.

To further investigate the correlation between GPD1 expression and breast cancer patient survival, a meta-analysis of the prognostic significance of GPD1 expression in human breast cancer was conducted using the Bc-GenExMiner v4.0 database. Univariate Cox analysis revealed that a low expression level of GPD1 was associated with poor metastatic relapse-free (MR-free) survival (Supplementary Table 1 and Figure 2A) (HR = 0.89, 95% CI: 0.83–0.96, *p* = 0.0013, *N* = 3,875) and any event (AE, metastasis or any relapse, or death) for patients (Supplementary Table 2 and Figure 2B (HR = 0.91, 95% CI: 0.86–0.96, *p* = 0.0005, *N* = 5,488). Further analysis using Kaplan-Meier curves with log-rank analysis for the overall survival of breast cancer patients was performed. Patients with low GPD1 expression (less than the median expression) had a significantly shorter survival time compared with patients with high GPD1 expression (greater than the median expression) (MR-free, *p* = 0.033, Figure 2C). Additionally, low levels of GPD1 mRNA were also significantly correlated with decreased survival time for AE patients (*p* = 0.04, Figure 2D).

### GPD1 is an independent marker of disease outcome in ER-positive and N-negative patients

**Figure 3:**
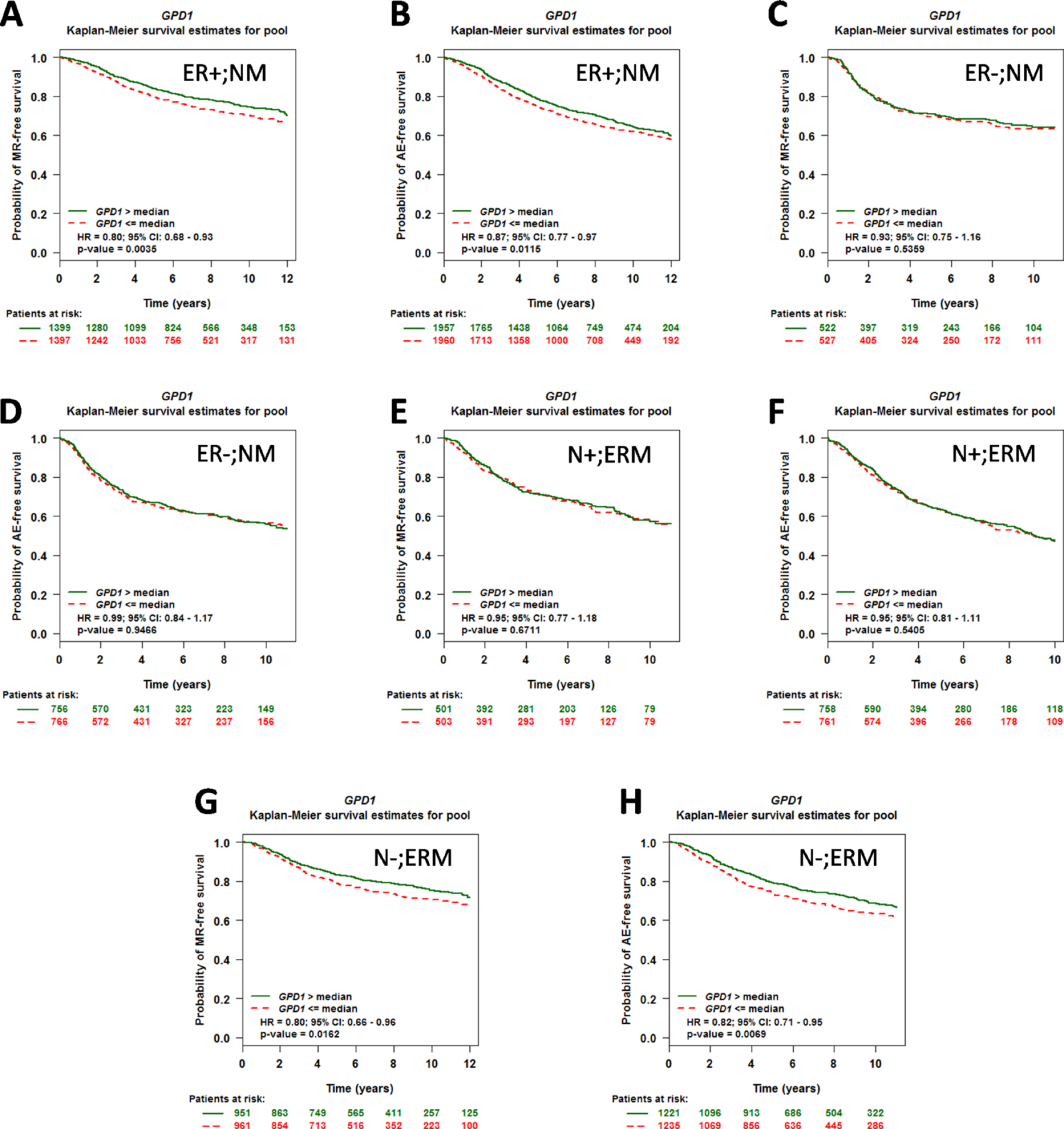
The prognostic impact of GPD1 on disease outcome in breast cancer patients with different ER and nodal statuses (**A**–**B**) Kaplan-Meier curves for GPD1 in patients with ER-positive status (*N* = 2,796 for MR and *N* = 3,917 for AE). (**C**–**D**) Kaplan-Meier curves for GPD1 in patients with ER-negative status (*N* = 1,049 for MR and *N* =1,522 for AE). (**E**–**F**) Kaplan-Meier curves for GPD1 in patients with nodal-positive status (*N* = 1,004 for MR and *N* = 1,519 for AE). (**G**–**H**) Kaplan-Meier curves for GPD1 in patients with nodal-negative status (*N* = 1,912 for MR and *N* = 2,456 for AE).

To assess the prognostic impact of GPD1 expression in patients with different ER or nodal statuses, a univariate Cox proportional hazards model analysis of each of the 18 possible pools corresponding to every combination of the population (nodal and ER status) and event criteria (MR or AE) was performed (Table 1). We found that the GPD1 expression level has significant prognostic value for breast cancer patients with ER-positive tumours (for NM, ER+, AE: *p* = 0.0027, HR = 0.91, 95% CI = 0.85–0.97, NP = 3,917, NE = 1,248 and for NM, ERM, AE: *p* = 0.0005, HR = 0.91, 95% CI = 0.86–0.96, NP = 5,488, NE = 1,854) (Supplementary Tables 3–4 and Supplementary Figure 3). However, in contrast, GPD1 expression did not have any significant prognostic value for ER-negative patients or for ER-positive and nodal-positive patients. The conflicting results suggest that the nodal status of breast cancer patients could also be a crucial factor for the prognostic significance of GPD1. In addition, nodal-negative patients also showed a significant correlation with GPD1 expression level (for ERM, N-, AE: *p* = 0.0013, HR = 0.87, 95% CI = 0.80–0.95, NP = 2,456, NE = 739) (Supplementary Table 5 and Supplementary Figure 4), which was consistent with a previous work [16]. Furthermore, we generated Kaplan-Meier curves based on the ER and nodal status. Low GPD1 expression levels correlated with both shorter MR-free and AE-free survival among the ER-positive patients (Figure 3A–3B) and nodal-negative patients (Figure 3G–3H), but not among ER-negative patients (Figure 3C–3D) and nodal-positive patients (Figure 3E–3F). The Kaplan-Meier curves based on the remaining 8 pools are shown in Supplementary Figure 5.

**Table 1:** Target prognostic analysis for the GPD1 expression levels in 18 pools corresponding to combinations of populations (ER and nodal status) and event criteria (MR or AE)

Nodal status	Estrogen receptor status	Event status	*p*-value	HR	95% CI	No. patients	No. events
NM	ERM	AE	0.0005	0.91	0.86–0.96	5488	1854
NM	ERM	MR	0.0013	0.89	0.83–0.96	3875	1010
N−	ERM	AE	0.0013	0.87	0.80–0.95	2456	739
NM	ER+	MR	0.0017	0.87	0.80–0.95	2796	670
NM	ER+	AE	0.0027	0.91	0.85–0.97	3917	1248
N−	ER+	MR	0.0052	0.83	0.73–0.95	1408	315
N−	ER+	AE	0.0056	0.87	0.78–0.96	1790	514
N−	ERM	MR	0.0067	0.86	0.78–0.96	1912	459
N+	ER+	AE	0.0653	0.9	0.80–1.01	1068	419
N+	ERM	AE	0.0737	0.92	0.83–1.01	1519	638
N+	ERM	MR	0.1882	0.92	0.81–1.04	1004	334
N−	ER−	AE	0.2193	0.91	0.78–1.06	641	220
N+	ER+	MR	0.2219	0.91	0.78–1.06	697	211
NM	ER−	AE	0.3714	0.96	0.87–1.05	1522	595
NM	ER−	MR	0.5181	0.96	0.84–1.09	1049	335
N−	ER−	MR	0.6733	0.96	0.79–1.16	485	142
N+	ER−	AE	0.7753	0.97	0.81–1.17	442	218
N+	ER−	MR	0.8453	0.98	0.77–1.24	299	122

N (+,−, m): nodal status (+: positive, −: negative, m: mixed); estrogen receptor status (+: positive, −: negative, m: mixed); HR: hazard ratio (values are rounded to 2 decimal places); 95% CI: 95% confidence interval (values were rounded to 2 decimal places); ER (+,−, m).

### Validation of the GPD1 expression in breast cancer tissues

**Figure 4:**
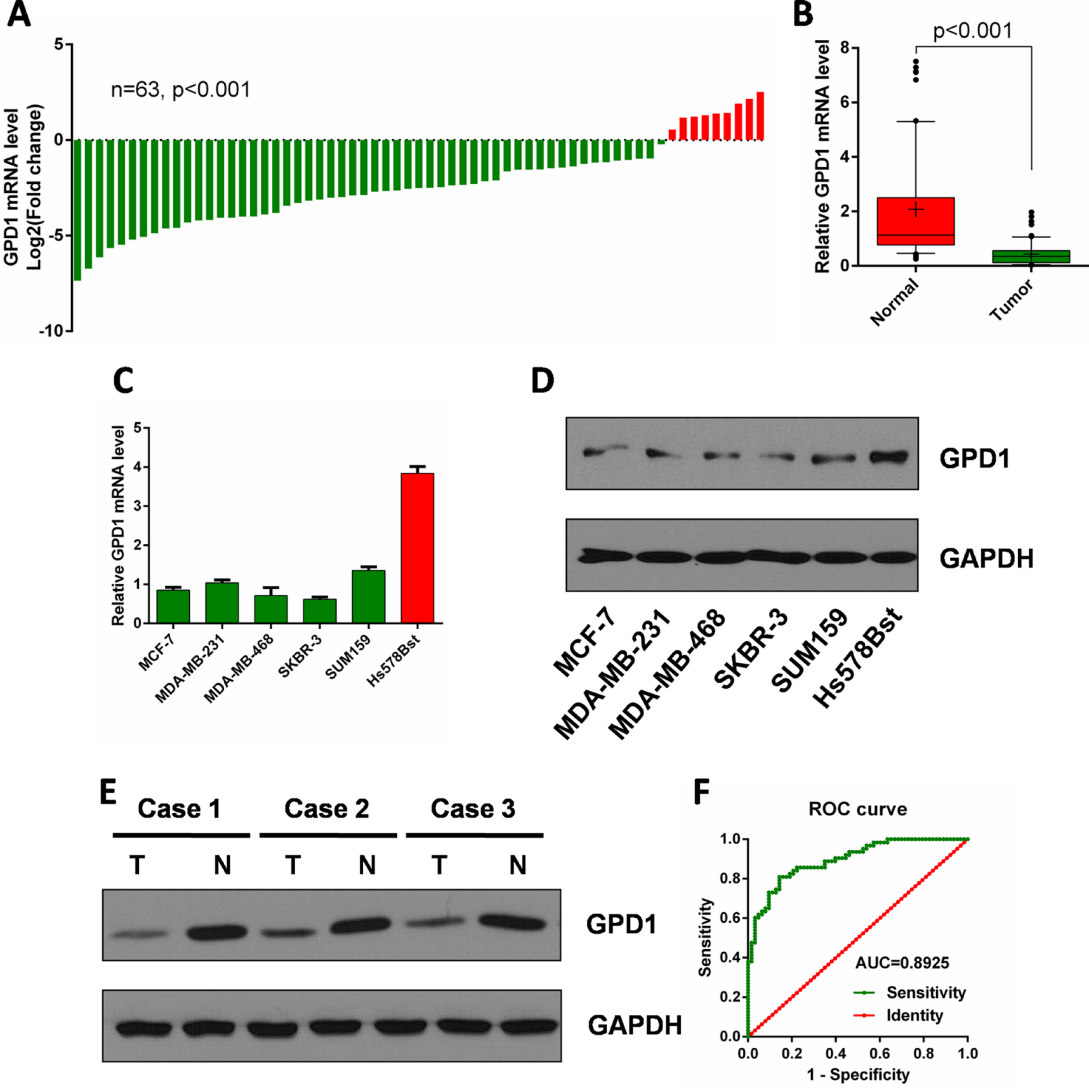
GPD1 is downregulated in human breast cancer (**A**) qRT-PCR analysis of GPD1 mRNA expression in 63 pairs of human breast cancer tissues and their corresponding adjacent normal tissues. GPD1 expression was normalized to GAPDH in each sample. (**B**) Box plot of GPD1 expression in human breast cancer tissues and adjacent normal tissues, Boxes represent interquartile ranges, and the horizontal lines across each box indicates the median values. (**C**) qRT-PCR analysis of GPD1 expression in human breast cancer cells (MCF-7, MDA-MB-231, MDA-MB-468, SKBR-3 and SUM159) and normal breast cells (Hs-578Bst). (**D**) Western blot analysis of GPD1 expression in human breast cancer cells (MCF-7, MDA-MB-231, MDA-MB-468, SKBR-3 and SUM159) and normal breast cells (Hs-578Bst). (**E**) Western blot analysis of GPD1 expression in 3 pairs of human breast tissues and their corresponding adjacent normal tissues. (**F**) The ROC curve of GPD1 expression in human breast cancer patients. Analysis of the GPD1 expression levels resulted in an area under the curve (AUC) value of 0.89, with a standard error of 0.027 and a 95% confidence interval of 0.84–0.95. Data are presented as the mean ± SD. Two-tailed Student's *t*-test was used.

To validate GPD1 expression in breast cancer at the mRNA level, qRT- PCR was performed on 63 paired surgical samples obtained from human breast cancer patients (cancerous tissues and the corresponding adjacent normal tissues from the same patients). The results showed that GPD1 was significantly downregulated in the majority of the paired human breast cancer tissues (54 out of 63) compared with the adjacent normal tissues (Figure 4A, *p* < 0.001). The median level of GPD1 mRNA expression was also significantly lower in the cancer tissues than in the adjacent normal tissues (Figure 4B, *p* < 0.001).

We also determined the mRNA expression levels of GPD1 in a normal breast cell line and five human breast cancer cell lines. GPD1 was significantly downregulated in human breast cancer cell lines (MCF-7, MDA-MB-231, MDA-MB-468, SKBR-3 and SUM159) when compared with a normal breast cell line (Hs-578Bst) (Figure 4C). Western blot analysis demonstrated that the protein level of GPD1 was also downregulated in human breast cancer cell lines when compared with a normal breast cell line (Figure 4D). We also found that GPD1 protein levels were significantly decreased in three pairs of human breast cancer tissues compared with normal tissues (Figure 4E).

To assess the potential of GPD1 as a diagnostic and prognostic marker of human breast cancer, we generated an receiver operating characteristic (ROC) curve and found that the GPD1 mRNA level in human breast cancer tissues substantially differs from that in control subjects, with an area under the curve (AUC) value of 0.89 (Figure 4F). Using the criterion value of 0.60, the sensitivity and specificity values were 0.81 and 0.86, respectively, to identify a patient with breast cancer, indicating that GPD1 serves as an excellent human breast cancer marker.

**Figure 5:**
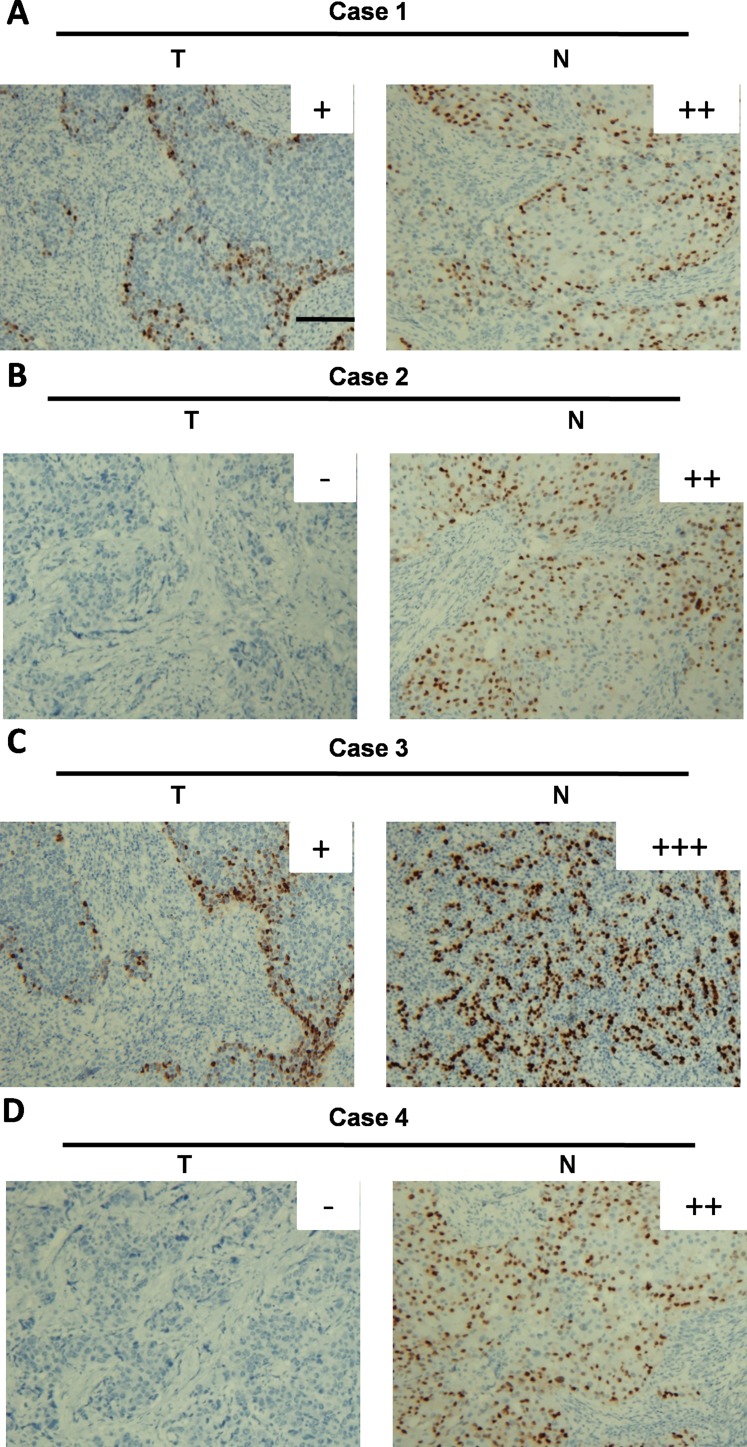
Immunohistochemical analysis of GPD1 protein expression in breast cancer tissues (**A**–**D**) The IHC images of four representative breast cancer tissue pairs are shown; GPD1 expression was scored as GPD1+ (A and C), GPD1++ (A, B and D), GPD1+++ (C) or GPD1- (B and D). Scale bar: 150 μm.

To consolidate our findings at the mRNA level, we performed IHC to investigate the protein level of GPD1 in all of the 63 paired breast cancer tissues which were used above. GPD1 protein was mainly observed both in the cytoplasm of cancer cells and the nucleus (Figure 5). In addition, consistent with the qRT-PCR results, IHC analyses showed that 42.86% (54/126) of the tissues exhibited low GPD1 expression (GPD1- or GPD1+) and 72/126 (57.14%) exhibited high GPD1 expression (GPD1++ or GPD1+++).

### Correlation between GPD1 expression and disease outcome in other human cancer types

**Figure 6:**
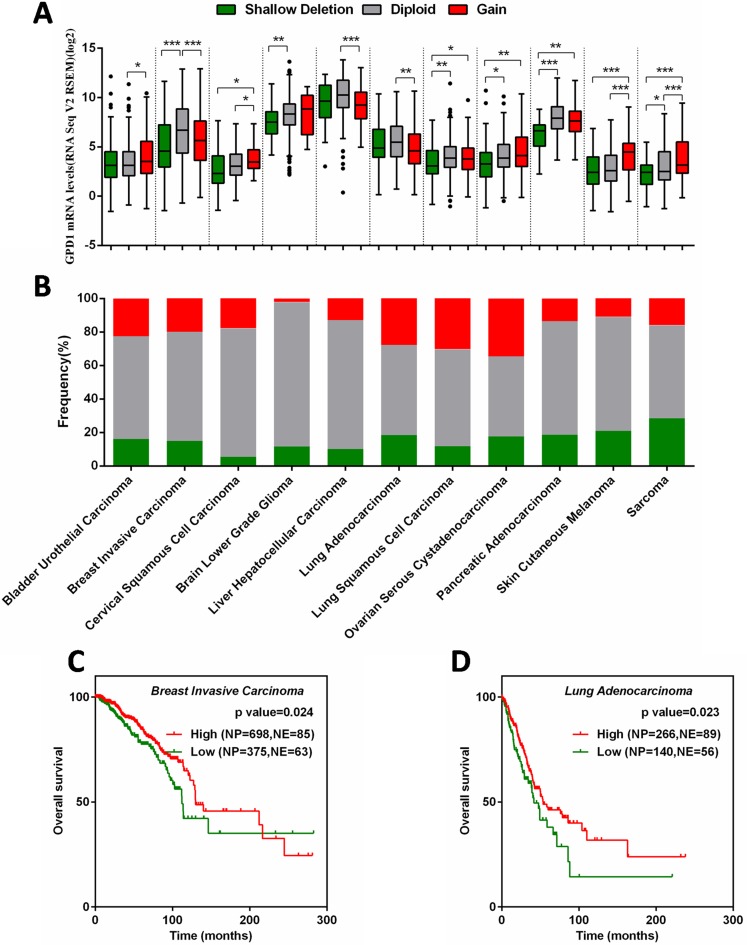
Correlation between GPD1 mRNA expression and disease outcome in several types of human cancers (**A**) Correlation between GPD1 mRNA expression levels and genomic alterations. (**B**) Frequencies of GPD1 genomic alterations in different types of human cancers. (**C**) Kaplan-Meier curves for GPD1 in breast invasive carcinoma patients. (**D**) Kaplan-Meier curves for GPD1 in lung adenocarcinoma patients. NP refers to Number of Patients; NE refers to Number of Events.

To investigate whether the downregulation of GPD1 was correlated with the pathogenesis of other cancers, 21 types of human cancers were chosen to assess the mRNA levels of GPD1 though the cBioPortal database. We found that the GPD1 copy number in the 21 human cancer types was different; it could be shallow deleted, unchanged (diploid) or gained, and 8 of the cancers exhibited deletion of GPD1 in more than 15% (38.1%, 8/21) of cases, while 2 exhibited deletion in more than 20% (9.52%, 2/21) of cases (Supplementary Table 7 and Figure 6B). The GPD1 mRNA levels were significantly downregulated in tumours with a shallow deletion of GPD1 compared to those without such changes, including breast invasive carcinoma, brain lower-grade glioma, lung squamous cell carcinoma, ovarian serous cystadenocarcinoma, pancreatic adenocarcinoma and sarcoma (*p* < 0.05, Figure 6A and Supplementary Figure 6), suggesting that GPD1 deletion results in reduced GPD1 mRNA expression in these cancers. However, seven cancer types with GPD1 amplification also exhibited reduced GPD1 mRNA expression levels (*p* < 0.05, Supplementary Table 7 and Figure 6A), suggesting additional mechanisms may lead to reduced mRNA levels of GPD1.

Kaplan-Meier analysis curves of the TCGA cohort were further applied in the 11 cancer types that exhibited reduced GPD1 mRNA expression level (Supplementary Figure 7). The results revealed that invasive breast carcinoma patients with reduced GPD1 mRNA have significantly reduced overall survival (*p* = 0.024, Figure 6C), which is consistent with our meta-analyses of the microarray datasets. Furthermore, the reduced GPD1 mRNA levels were also correlated with decreased overall survival in lung adenocarcinoma patients (*p* = 0.023, Figure 6D). These findings suggest that GDP1 is a tumour-suppressor gene in human breast cancer and lung adenocarcinoma.

### GPD1 inhibits breast cancer cell proliferation, migration, and invasion

**Figure 7:**
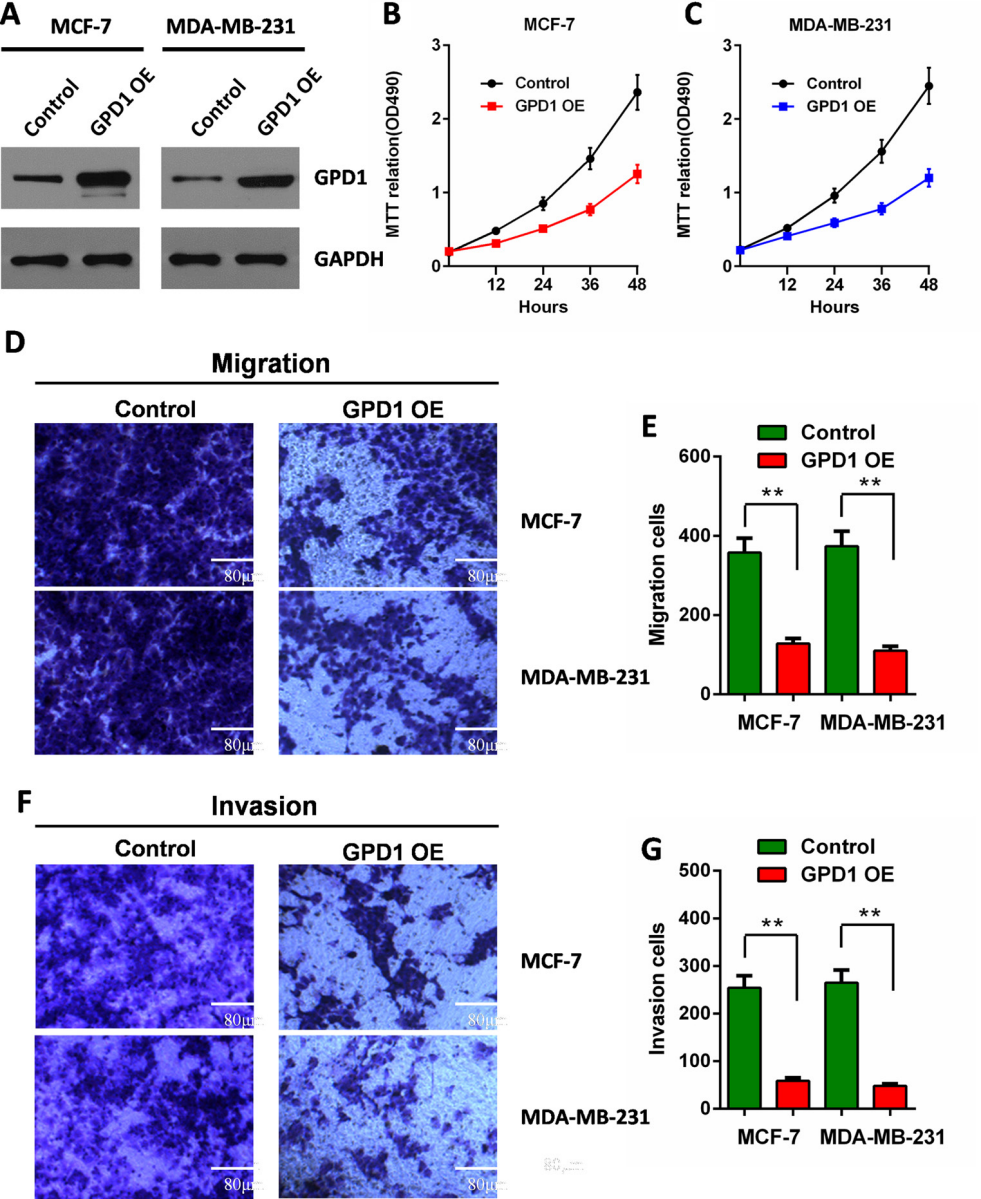
GPD1 inhibits human breast cancer cell proliferation, migration and invasion (**A**) The expression levels of GPD1 after transfection of MCF-7 and MDA-MB-231 cells with a recombinant GPD1 expression plasmid; GAPDH was used as a control. (**B**) MTT assays indicated the cell proliferation for MCF-7 cells. (**C**) MTT assays indicated the cell proliferation for MDA-MB-231 cells. (**D**–**G**) Transwell assays show the effects of GPD1 on MCF-7 and MDA-MB-231 breast cancer cell migration (D–E) and invasion (F–G). The data are presented as the mean values ± SD. A two-tailed Student's *t*-test was used. ^*^p < 0.05, ^**^p < 0.01.

To examine the effects of GPD1 on the proliferation of the human breast cancer cell lines. MCF-7 and MDA-MB-231cells were transfected with the recombinant GPD1-expressing plasmid to increase the expression level of GPD1.Western blotting analysis was used to verify the expression efficiency of GPD1 (Figure 7A). MTT assays were further performed, and as expected, transfection with the GPD1 expression plasmid decreased the proliferation of MCF-7 breast cancer cells compared with control cells which transfected with empty vector. Consistent results were also observed in MDA-MB-231 cells (Figure 7B–7C).

We further evaluated migration and invasion of human breast cancer cells by examining the effects of exogenous GPD1. The effect of GPD1 on cell migration was determined using Transwell assays. We found that cells expressing exogenous GPD1 exhibited a significantly decreased migratory ability compared with cells transfected with empty vector (Figure 7D–7E). To examine the effect of GPD1 on cell invasion, we cultured MCF-7 and MDA-MB-231 cells expressing exogenous GPD1 in Transwell chambers pre-coated with Matrigel for 24 hours prior to measurements. We found that increased GPD1 expression significantly decreased the ability of the cells to cross the Matrigel-coated inserts (Figure 7F–7G).

### GPD1 is a direct target of miR-370

**Figure 8:**
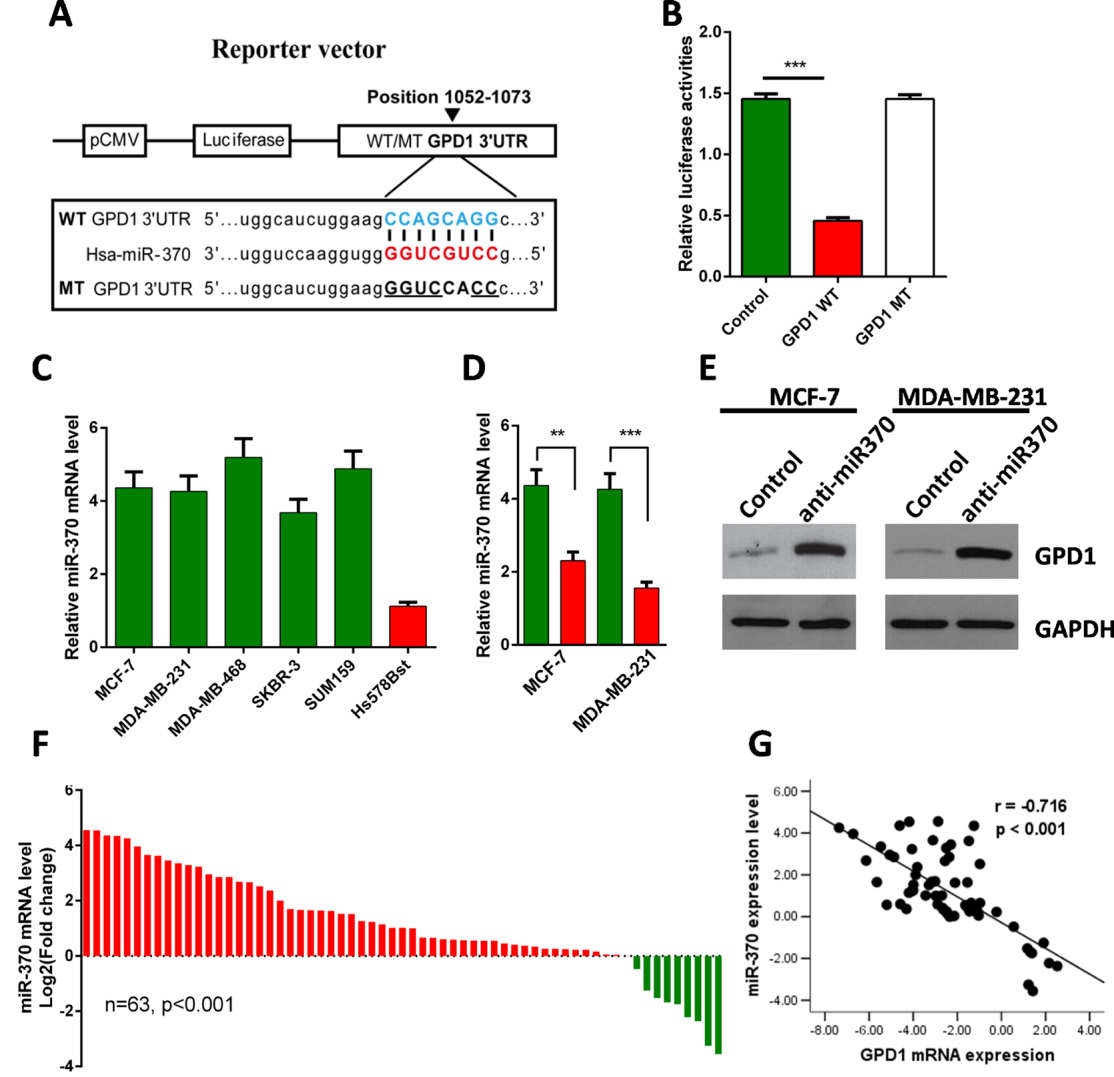
GPD1 is a direct target of miR-370 (**A**) Diagrammatic representation of miR-370 and its putative binding sequence in the 3′-UTR of GPD1 and the luciferase reporter plasmids with WT and MT GPD1 3′-UTRs. (**B**) Relative luciferase activity in MCF-7 cells after transfection with WT or MT GPD1 3′-UTR plasmid and co-transfection with miR-370 inhibitors. (**C**) The relative miR-370 mRNA levels in MCF-7, MDA-MB-231, MDA-MB-468, SKBR-3, SUM159 and normal breast cells (Hs-578Bst). (**D**–**E**) miR-370 inhibitors facilitated the expression of GPD1 both at the mRNA (D) and protein level (E) in MCF-7 and MDA-MB-231 cells. GAPDH was used as a control. (**F**) qRT-PCR analysis of miR-370 mRNA expression in 63 pairs of human breast cancer tissues and their corresponding adjacent normal tissues. miR-370 expression was normalized to U6 snRNA expression in each sample. (**G**) Inverse correlation between miR-370 and GPD1 expression in human breast cancer tissues. Data are presented as the mean ± SD. Two-tailed Student's *t*-test was used. ^*^p < 0.05, ^**^p < 0.01, ^***^p < 0.001.

miRNAs have been shown to be profoundly involved in the pathogenesis of many human cancers [17, 18]. In addition, miR-370 was recently discovered to be upregulated in human breast cancer cells and was shown to be significantly correlated with breast cancer progression [19]. From the results of multiple prediction algorithms by Targetscan, PicTar and miRanda, we queried the possibility that GPD1 was a direct target of miR-370, and then, a luciferase reporter assay was performed (Figure 8A). The results demonstrated that miR-370 significantly repressed the activity of reporter vectors harbouring the wild-type 3′-UTR of GPD1 (GPD1 WT), whereas mutations of putative miR-370 binding sites in the 3′-UTR (GPD1 MT) abrogated the inhibitory effects of miR-370 in MCF-7 cells (Figure 8B).

To further investigate the relationship between GPD1 and miR-370, we analysed the expression of miR-370 in breast cell lines and 63 paired breast cancer tissues. We found higher levels of miR-370 in human breast cancer cell lines (MCF-7, MDA-MB-231, MDA-MB-468, SKBR-3 and SUM159) compared with the normal breast cell line (Hs-578Bst) (Figure 8C), and western blot analyses showed that protein levels of GPD1 were dramatically upregulated in MCF-7 and MDA-MB-231 cells when miR-370 expression was inhibited (Figure 8D-E). We also found that miR-370 was significantly upregulated in the majority of paired human breast cancer tissues compared with the adjacent normal tissues (Figure 8F, *p* < 0.001), and the miR-370 levels were inversely correlated with GPD1 levels (*r* = −0.716, *p* < 0.001, Figure 8G). Taken together, the results suggest that miR-370 directly suppress GPD1 expression in human breast cancer.

## DISCUSSION

In this study, we first identified the expression level of GPD1 using TCGA and Oncomine databases, and further evaluated the prognostic value of GPD1 expression in human breast cancer through meta-analysis of public microarray profiles. Our results indicate that breast cancer patients have significantly lower expression levels of GPD1 compared with normal controls, which was further verified in 63 paired human breast cancer tissues (54 out of 63) compared with the adjacent normal tissues. In addition, low GPD1 mRNA levels are associated with decreased overall and MR-free survival time, particularly in ER-positive and nodal-negative patients. We also assessed the prognostic utility of GPD1 expression in predicting disease outcomes within the individual molecular subtypes. GPD1 expression levels among the HER2-E subtype (HER2-enriched) tumours were correlated with a more favourable prognosis compared to the other four subtypes based on Sorlie's [20] (*p* = 0.0051, HR = 0.75, 95% CI = 0.61–0.92, NP = 595) and Hu's [21] (*p* = 0.0386, HR = 0.79, 95% CI = 0.64–0.99, NP = 485) classifications (Supplementary Table 6). In addition, the results of Kaplan-Meier curve analysis revealed that HER-E subtype patients with low GPD1 levels have reduced overall survive time , although the results were not significant (Supplementary Figure 8).

Notably, we further found that exogenous expression of GPD1 in human MCF-7 and MDA-MB-231 breast cancer cell lines significantly inhibited cell proliferation, migration and invasion. We previously reported that PLIN1, a core component of lipid drops that regulates both triglyceride storage and lipolysis, is involved in breast cancer progression though PLIN1-mediated lipid metabolism [22]. Combing the crucial role of GPD1, which connects carbohydrate and lipid metabolism, with the reported altered metabolism that occurs in the malignant transformation of cells [23–25] and the specific adaptations in anabolic pathways that supply rapidly proliferating cells with the building blocks needed to produce nucleic acids, proteins and lipids, driving the formation of biomass [26, 27], suggests that the reduced GPD1 level possibly limited the conversion of G3P to DHAP and thus caused an increase in the amount of G3P available for TG synthesis, which was then used for producing energy. Alternatively, there could be another correlation between GPD1 and PLIN1 in the progression of breast cancer.

It has been reported that the expression level of GPD1 is regulated by dexamethasone in a glucocorticoid receptor (GR) dependent way in hepatocarcinoma cells [28]. However, the transcriptional and post-transcriptional regulation of the GPD1 gene is largely unknown. Here, we found that the expression of GPD1 was inhibited by miR-370. Deregulation of miR-370 has been reported in various cancers, in which it can act as either an oncogene [29] or a tumour-suppressor gene [30, 31]. However, the expression of miR-370 in breast cancer was reported to be increased, which was the same as our results in the 63 paired human breast cancer tissues. miR-370 was reported to be upregulated and to function as an oncogene by targeting FoxO1 in human prostate and gastric cancers [32, 33]. Combining the regulation of FoxO1 and PGC1α with glucose metabolism [34], it is worth further study to identify the relationship between FoxO1, PGC1α and GPD1.

In conclusion, the current study identified correlations between GPD1 expression in breast cancer and highlights the prognostic value of GPD1 mRNA levels in breast cancer. These results indicated that low levels of GPD1 are linked to tumour progression and worse disease-free survival, and GPD1 acts as a tumour-suppressor gene. Although further studies are needed to clarify the precise mechanism of the tumour-suppresser effect of GPD1 in the development and progression of breast cancer, understanding the role of GPD1 may provide the basic knowledge required for the development of potential prognostic biomarkers and targeted therapies.

## MATERIALS AND METHODS

### Ethics statement

All specimen collections and the study protocol were approved by the Ethics Committee of Hubei Cancer Hospital on June 24th 2015. The study was performed according to the tenets of the Declaration of Helsinki. All patients provided written informed consent before participating in this research.

### Cell culture and clinical specimens

The human breast cancer cell lines MCF-7, MDA-MB-231, MDA-MB-468, SKBR-3 and SUM159 were purchased from the Cell Center of the Institute of Biochemistry and Cell Biology, Chinese Academy of Sciences (Shanghai, China). The cells were cultured in Dulbecco's modified Eagle's medium (DMEM) (Cat# 11965, Gibco, USA) supplemented with 10% foetal bovine serum (Cat# 10100, Gibco, USA), 100 U/ml penicillin G and 100 μg/ml streptomycin at 37°C in a humidified incubator containing 5% CO_2_. A total of 63 pairs of human breast cancer tissues (cancerous tissues and the corresponding adjacent normal tissues from the same patients) were collected from Hubei Cancer Hospital (Hubei, China) between 2013 and 2015 during surgery and made into paraffin sections (4 μm), No enrolled patients underwent radiation or chemotherapy prior to surgery. All patients were histologically confirmed, and tumoural samples were checked to ensure that tumoural tissue was present in more than 80% of the specimens.

### Expression vector construction

The GPD1 expression vector and control vector were constructed and cloned in between the 5′ EcoR I and 3′ Bam HI sites of a p3xflag-cmv-10 vector (Cat# E7658, Sigma, USA) according to the manufacturer's instructions. Commercially synthesized 2’ -O-methyl-modified antisense oligonucleotide of miR-370 was used as a miR-370 inhibitor (anti-miR-370). The sequence of anti-miR-370 was 5′ - GTAACTGCAGAGACGTGACCTG -3′. Lipofectamine 2000 Transfection Reagent (Cat# 11668, Invitrogen, Carlsbad, USA) was used to transfect the MCF-7 and MDA-MB-231 cell lines with the GPD1 expression vector according to the manufacturer's instructions.

### The Cancer Genome Atlas (TCGA) data analysis

Breast cancer UNC IlluminaHiSeq_RNASeq Level 3 data were downloaded from The Cancer Genome Atlas Data Portal (https://tcga-data.nci.nih.gov/tcga/) maintained by the National Cancer Institute and National Human Genome Research Institute. The calculated expression was for all reads aligning to a particular gene per sample. Data from a total of 30 breast cancer patients were available for gene expression analysis. The RNAseq data were grouped into Tumour tissues (*n* = 20) and Normal tissues (*n* = 10) based on TCGA annotation. The heatmap analysis of the gene expression pattern was performed using R version 3.2.2 software for Windows with “DESeq” and “edgeR” packages. Genes were hierarchically clustered using complete linkage and Euclidian distance. Fold-change analysis was performed on the two categories of samples (Normal and Tumour), followed by an unpaired *t*-test (unequal variance) that was performed to obtain significant gene entities. A *p*-value computation (asymptotic) was further performed to obtain gene entities with *p* < 0.01.

### RNA extraction and qRT-PCR

RNA was extracted using Ambion^®^ RNA extraction kit (Cat# 10928-034, Life Technologies, USA) according to the manufacturer's instructions. Then, RNA was reverse transcribed and amplified by qRT-PCR using a SuperScript^®^ One-Step RT-PCR System with Platinum^®^ Taq DNA Polymerase (Cat# 10966034, Life Technologies, USA) on a 7500 Real Time PCR System (Applied Biosystems, USA). The annealing and extension steps were separated as follows (three-step cycling): Step 1, 1 cycle at 45–55°C for 20 minutes plus 94°C for 2 minutes; step 2, 35 cycles of denaturing at 94°C for 15 seconds, annealing at 55–60°C for 30 seconds and extending at 68–72°C for 1 minute/kb. The RNA expression level for each sample was normalized to the expression of GAPDH or RNU6B and calculated using the 2^-ΔΔct^ method [35], with three biological replicates of comparative qRT-PCR. The following primer sequences were used for qRT-PCR: GPD1, (forward) 5′- TGCTGAATGGGCAGAAAC-3′ and (reverse) 5′- AAAT GTGGTGGCATGAGG-3′; GAPDH, (forward) 5′- AGCCACATCGCTCAGACAC -3′ and (reverse) 5′- GCCCAATACGACCAAATCC -3′; miR-370-RT, 5′- GTCGTATCCAGTGCAGGGTCCG AGGTGCACTGGATACGACACCAGG -3′, (forward) 5′- TGCGGGCCTGCTGGGGTGGAAC -3′ and (reverse) 5′-CCAGTGCAGGGTCCGAGGT -3′; RNU6B-RT, 5′- GTCGTATCCAGTG CAGGGTCCGAGGTATTCGCACTGGATACGACAA AATATGGAAC -3′, (forward) 5′- TGCGGG TGCTCGCTTCGGCAGC -3′ and (reverse) 5′- CCA GTGCAGGGTCCGAGGT -3′.

### Western blotting

Total protein from MCF-7, MDA-MB-231, MDA-MB-468, SKBR-3 and SUM159 cell lysates were extracted by resuspending the cell pellets in RIPA buffer (150 mM NaCl, 50 mM Tris (pH 7.4) and 1% Triton X-100). The protein concentration was measured using a BCA Protein Assay Kit (Cat# 23227, Thermo, USA). Approximately 30 μg of total protein per sample was separated by SDS-PAGE and then transferred onto nitrocellulose membranes. Western blot analyses were performed with polyclonal antibodies against GPD1 (Cat# sc-376219, Santa Cruz Biotechnology, USA), with a monoclonal GAPDH antibody as a control (Cat# G9545, Sigma, USA).

### Immunohistochemistry

Immunohistochemistry was performed as previously described [36]. Briefly, paraffin sections were deparaffinized and then rehydrated for 10 minutes. Hydrogen peroxide (0.3% v/v) was then applied to block endogenous peroxide activity, and the samples were microwave heated in 15 μM citrate buffer (pH 6.0) for 3 minutes to expose the antigens. The tissue sections were incubated with a GPD1 polyclonal antibody (1:1,000 dilution, Santa Cruz Biotechnology) after incubation with normal goat serum. Next, the samples were incubated with the secondary biotinylated goat anti-rabbit serum immunoglobulin G (IgG) antibody at 37°C for 30 minutes followed by antibody staining overnight at 4°C. After washing, the paraffin sections were incubated with streptavidin-avidin-conjugated horseradish peroxidase for 30 minutes. Counterstaining with haematoxylin was performed for 30 minutes, and the paraffin sections were dehydrated in ethanol prior to mounting. To quantify the status of GPD1 protein expression in those components, an IHC scoring systems was used as described previously [37]. The intensity of the GPD1 immunoreaction was scored as follows: -, none; +, weak; ++, moderate; and +++, intense. Based on the GPD1 expression levels, the breast cancer patients were divided into two groups: a low GPD1 expression group (GPD1- or GPD1+) and a high GPD1 expression group (GPD1++ or GPD1+++).

### Cell proliferation assay

Cell proliferation assays were performed as previously described [36]. Briefly, cells (1 × 10^5^ cells/well) were seeded into 6-well plates. Cell proliferation was examined at 12, 24, 36 and 48 hours after transfection. The cells were stained at the indicated time points with 100 μl of sterile MTT dye (0.5 mg/ml, Cat# M2128, Sigma, USA) for 4 hours at 37°C, followed by removal of the culture medium and the addition of 150 μl of DMSO (Cat# D8418, Sigma). The number of viable cells was assessed by measurement of the absorbance at 490 nm. All experiments were performed in triplicate. Cell migration and invasion assays.

### Cell migration and invasion assays

Cell migration and invasion assays were performed as previously described [22]. Briefly, for cell migration assays, 1 × 10^5^ cells were seeded onto the upper transwell chambers (Cat# 3452, Costar Corning, USA). and then they were all incubated for 24 h at 37°C. The lower chamber was added with 500 μL DMEM supplemented with 10% fetal bovine serum as a chemoattractant. Then the inserts were washed with phosphate buffered saline and the cells on the top surface of the inserts were removed with a cotton swab. Cells adhering to the lower insert membrane surfaces were fixed with methanol stained with crystal violet solution and quantified using ImageJ software. All assays were independently repeated in triplicates. The procedure for cellular invasion assays was similar to that of the cell migration assays, except that the transwell membranes were precoated with 24 μg/μl matrigel.

### Dual-luciferase reporter assay

The 3′UTR of GPD1 mRNA amplified from normal breast tissue genomic DNA by PCR was constructed and cloned in between the SacI and XhoI sites of a pmirGLO Dual-Luciferase miRNA Target Expression Vector (Cat# E1330, Promega). The primer sequences used for GPD1 3′UTR qRT-PCR were (forward) CCAGAGGACATCCCTGACTCC and (reverse) GTTCATCAGCCTTTGGGCC. For the luciferase reporter gene assay, 5 × 10^5^ MCF-7 cells were seeded in triplicate in 24-well plates and 80 ng of wild-type or mutant reporter constructs (pGL3 GPD1-3′UTR-WT or pGL3 GPD1-3′UTR-MT) were transfected into MCF-7 cells, along with Renilla plasmid, using lipofectamine 2000 (Invitrogen). Twenty-four hours after transfection, cells were lysed, and luciferase activities were determined as for a dual-luciferase assay reporter system according to the manufacturer's instructions.

### Statistical analysis

ROC curve analysis was used to evaluate the predictive power of each biomarker. AUC was computed via numerical integration of the ROC Curves. The median cut was used in all survival analyses and log rank *p*-values were calculated. The difference in GPD1 mRNA expression levels between different statuses, PAM50 subtype, age, SBR and NPI in bc-GenExMiner v4.0 was analysed using a Dunnett-Tukey-Kramer test. Group comparisons were performed using a Mann-Whitney test, and the two-tailed *p*-value is shown. Survival analysis and ROC curves were performed using GraphPad Prism version 6.01. Cox proportional hazard model analysis was used to calculate hazard ratios and to identify factors affecting survival. All analyses were performed using SPSS 13.0 for windows. A two-tailed *p*-value of less than 0.05 was considered statistically significant.

## SUPPLEMENTARY MATERIALS FIGURES AND TABLES


