# Supplementary material for: Identification of glycerol-3-phosphate dehydrogenase 1 as a tumour suppressor in human breast cancer
Source: Oncotarget. 2017 Sep 19;8(60):101309–24. doi: 10.18632/oncotarget.21087 (PMC5731876; doi:10.18632/oncotarget.21087)
Supplement: Supplementary file 1 [file oncotarget-08-101309-s001.pdf]

Identification of glycerol-3-phosphate dehydrogenase 1 as a tumour suppressor in human breast cancer

SUPPLEMENTARY MATERIALS

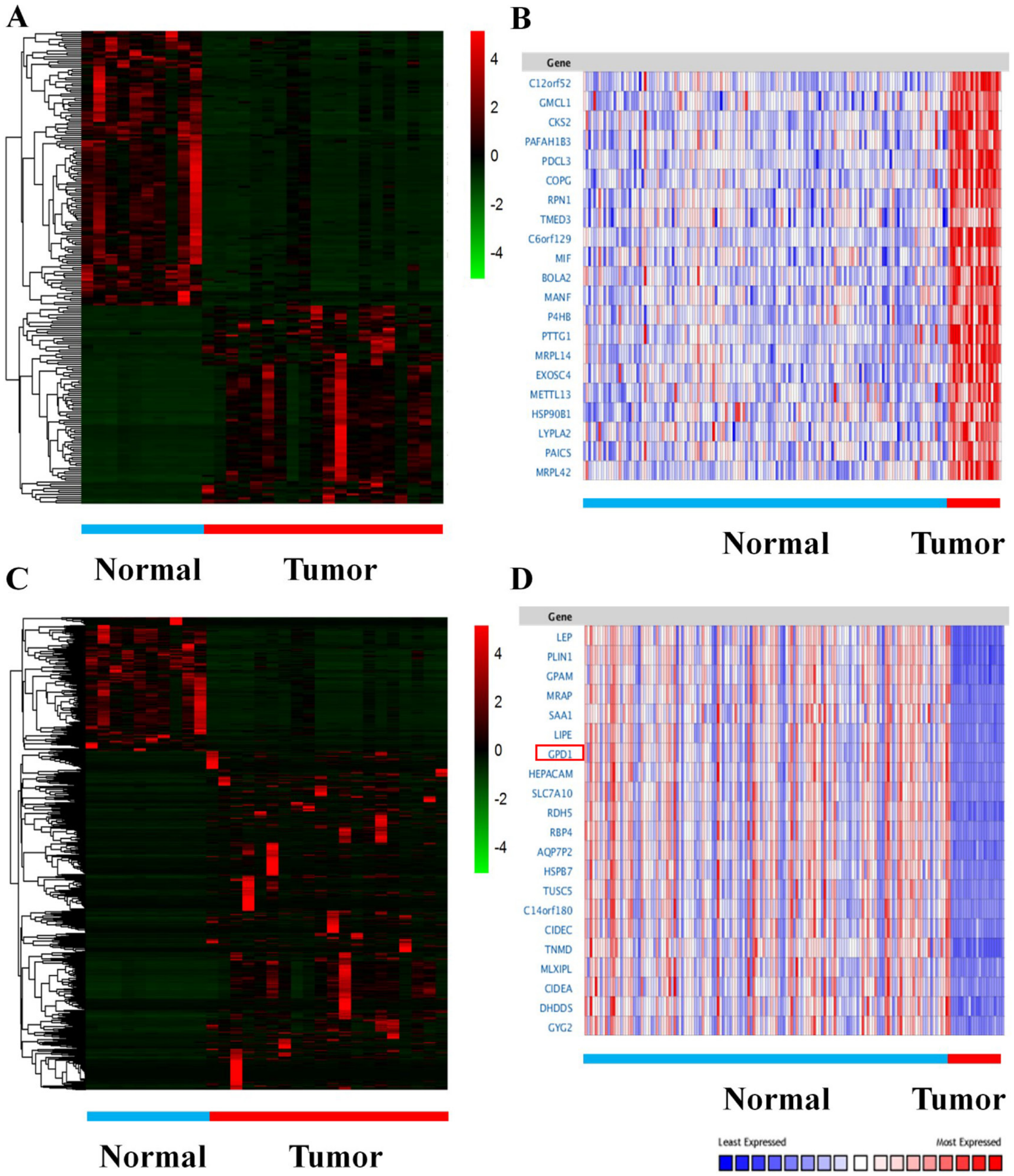

Supplementary Figure 1: Heatmap illustrating genes expression profiles for the 30 breast cancer cases (10 normal and 20 tumors) performed by R version 3.2.2 software with DESeq package ($p < 0.01$ and log2 Fold Change > 3) (**A**) and edgeR package ($p < 0.01$ and log2 Fold Change > 3) (**C**). Heatmap illustrating genes expression profiles for the top 21 up regulated (**B**) and down regulated (**D**) genes in human breast cancer. Red frame indicate the expression of GDP1.

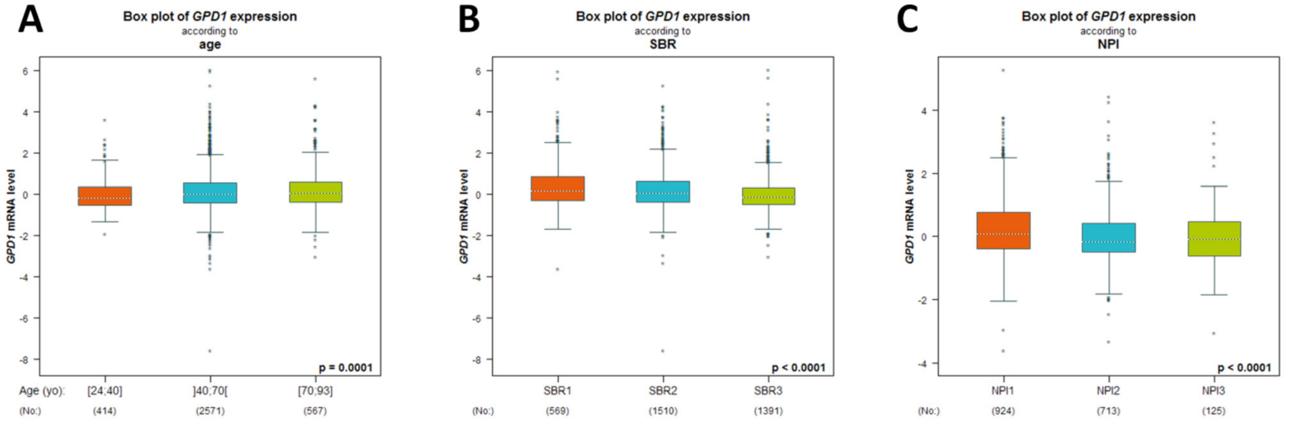

D

p(Welch) = 0.0001

**Dunnett-Tukey-Kramer's test:**	
groups comparison	p-value
[70;93] > [24;40]	< 0.001
]40;70[> [24;40]	< 0.01
[70;93] =]40;70[	> 0.10

E

p(Welch) < 0.0001

**Dunnett-Tukey-Kramer's test:**	
groups comparison	p-value
SBR2 < SBR1	< 0.0001
SBR3 < SBR1	< 0.0001
SBR3 < SBR2	< 0.0001

F

p(Welch) < 0.0001

**Dunnett-Tukey-Kramer's test:**	
groups comparison	p-value
NPI2 < NPI1	< 0.0001
NPI3 < NPI1	< 0.05
NPI3 = NPI2	> 0.10

Supplementary Figure 2: The expression of GPD1 in human breast cancer patients with different age (*N* = 3,552, **A**, **D**), scarf-bloom-richardson grading (SBR, *N* = 3,470, **B**, **E**) and Nottingham prognostic index (*N* = 1,762, **C**, **F**).

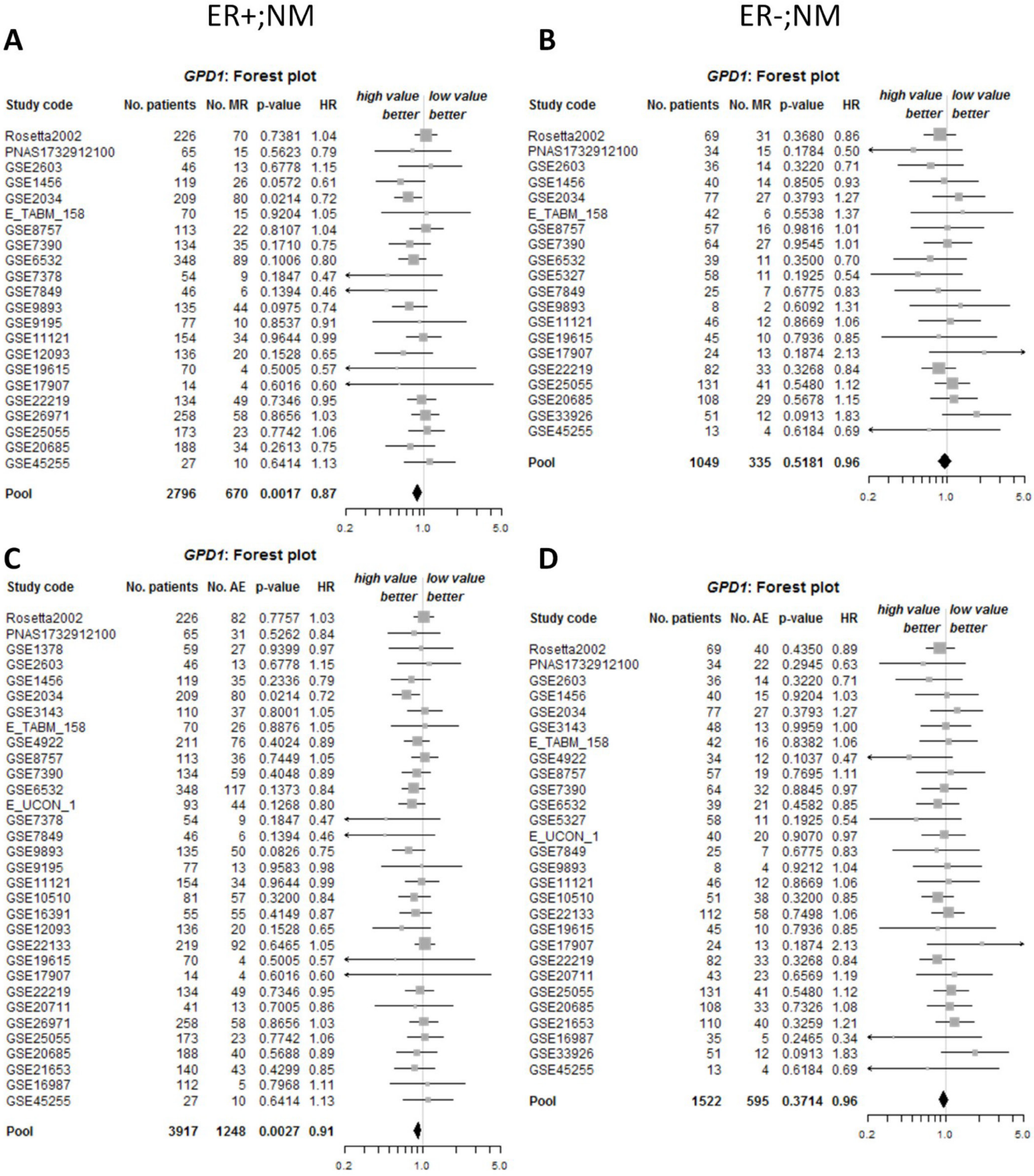

Supplementary Figure 3: Forest plot for GPD1 with mixed nodal status and positive ER status patients with metastatic relapse (**A**), any event information (metastatic or any relapse, or death) (**C**). Forest plot for GPD1 with mixed nodal status and negative ER status patients with metastatic relapse (**B**), any event information (metastatic or any relapse, or death) (**D**).

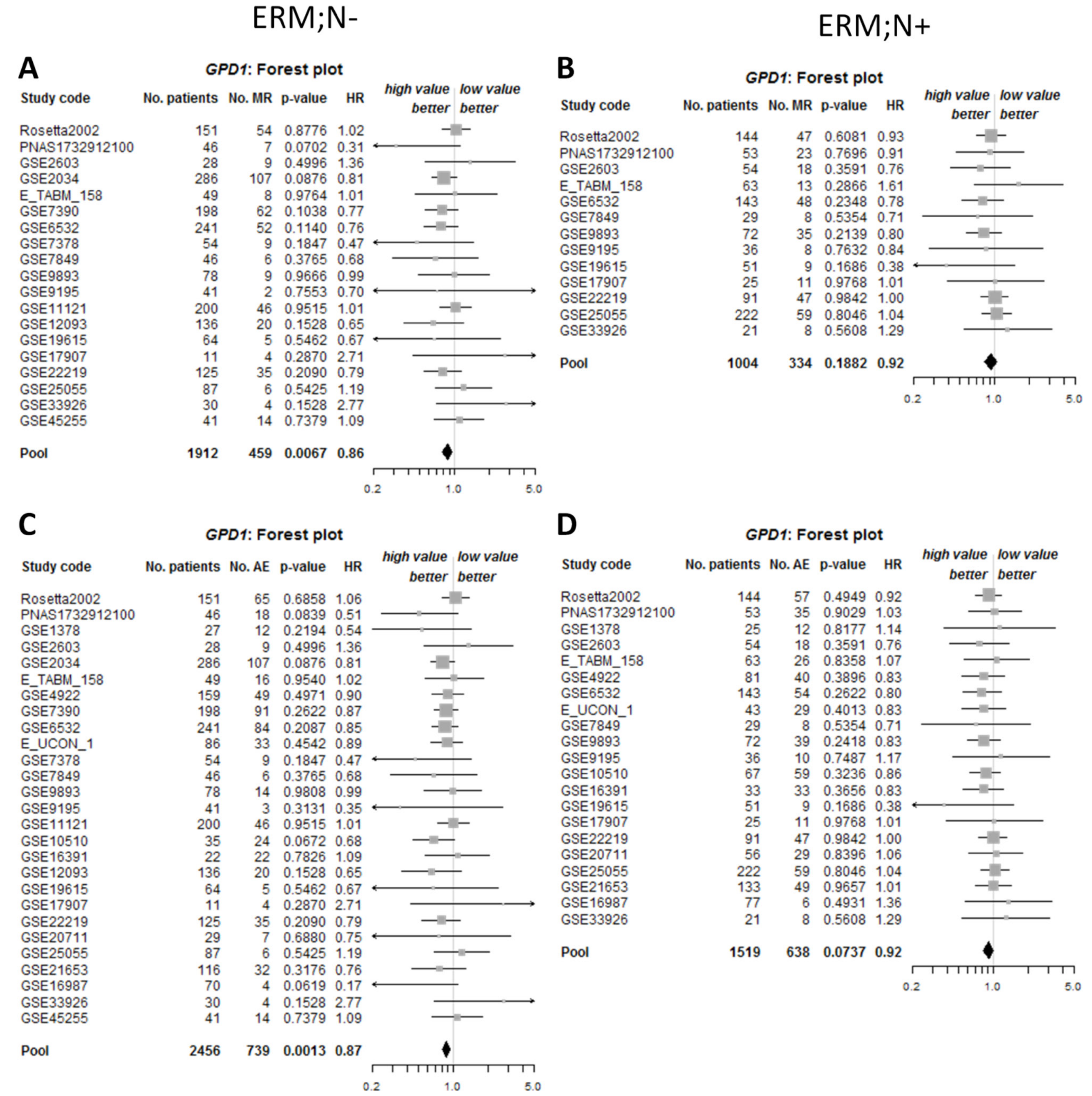

Supplementary Figure 4: Forest plot for GPD1 with mixed ER status and negative nodal status patients with metastatic relapse (**A**), any event information (**C**). Forest plot for GPD1 with mixed ER status and positive nodal status patients with metastatic relapse (**B**), any event information (**D**).

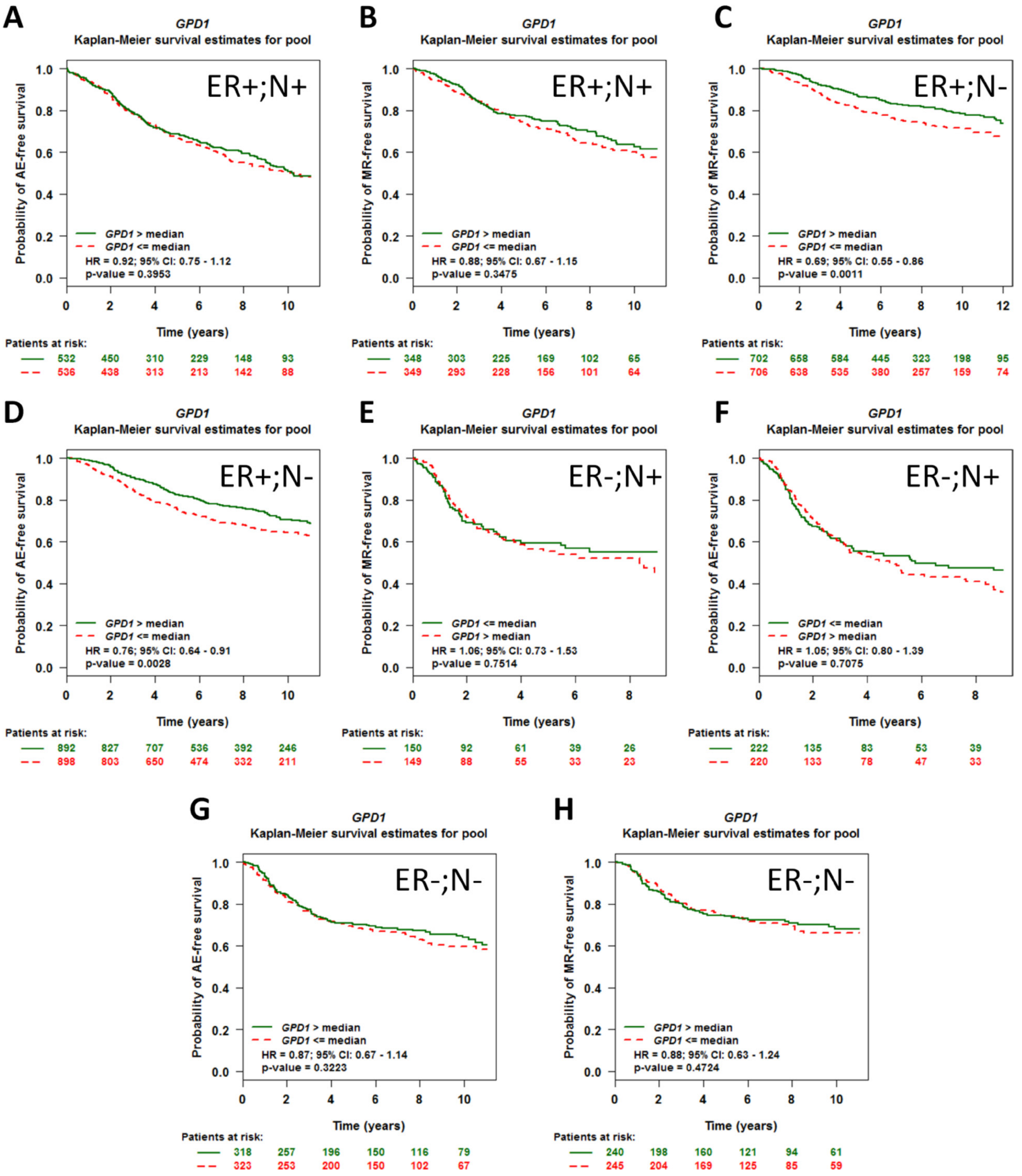

Supplementary Figure 5: Kaplan-Meier curves based on the remaining 8 pools corresponding to every combination of population (nodal and ER status) and event criteria (MR or AE).

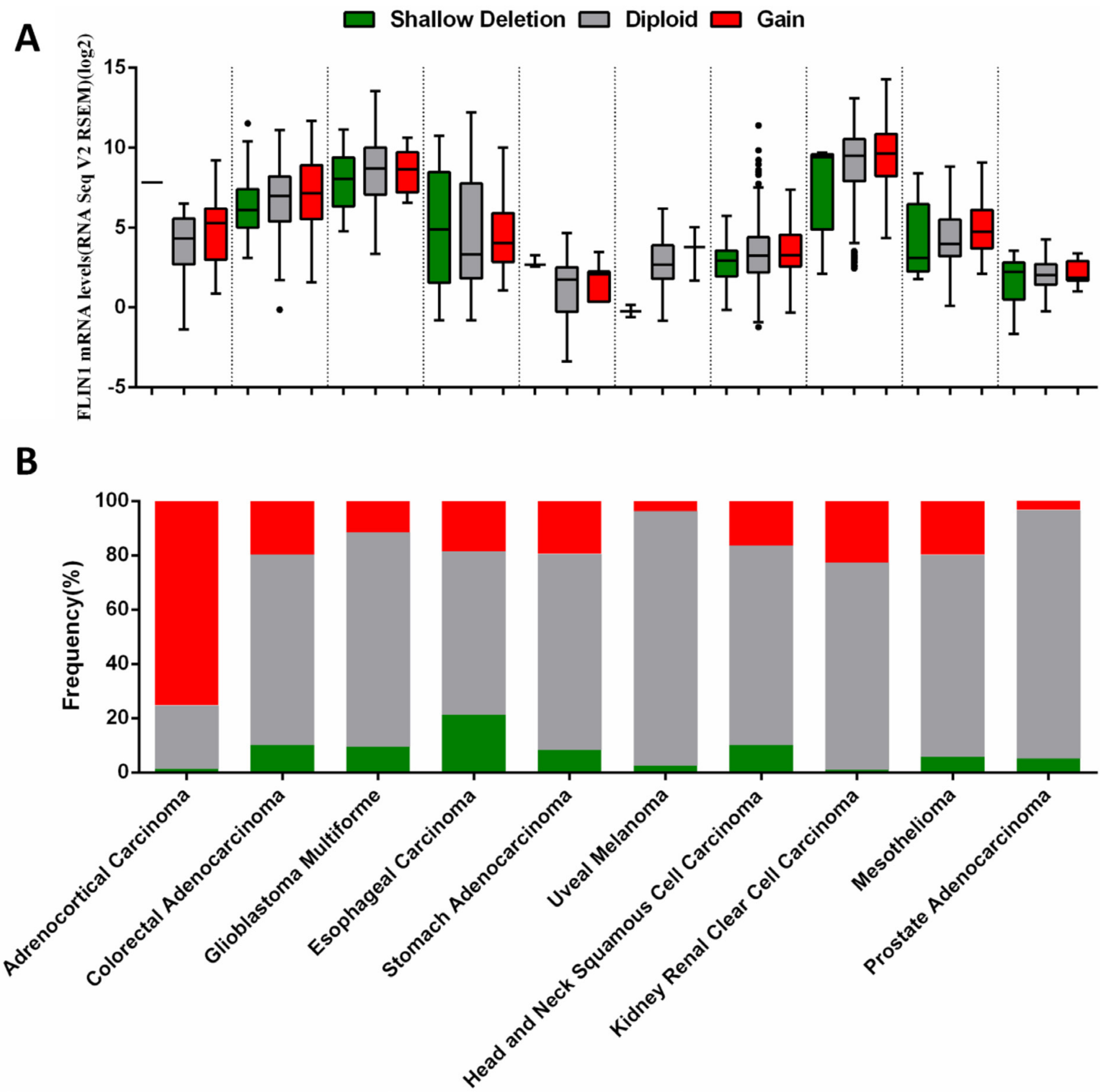

Supplementary Figure 6: (**A**) Correlation between GPD1 mRNA expression levels and genomic alterations. (**B**) Frequencies of GPD1 genomic alterations in different human cancer types.

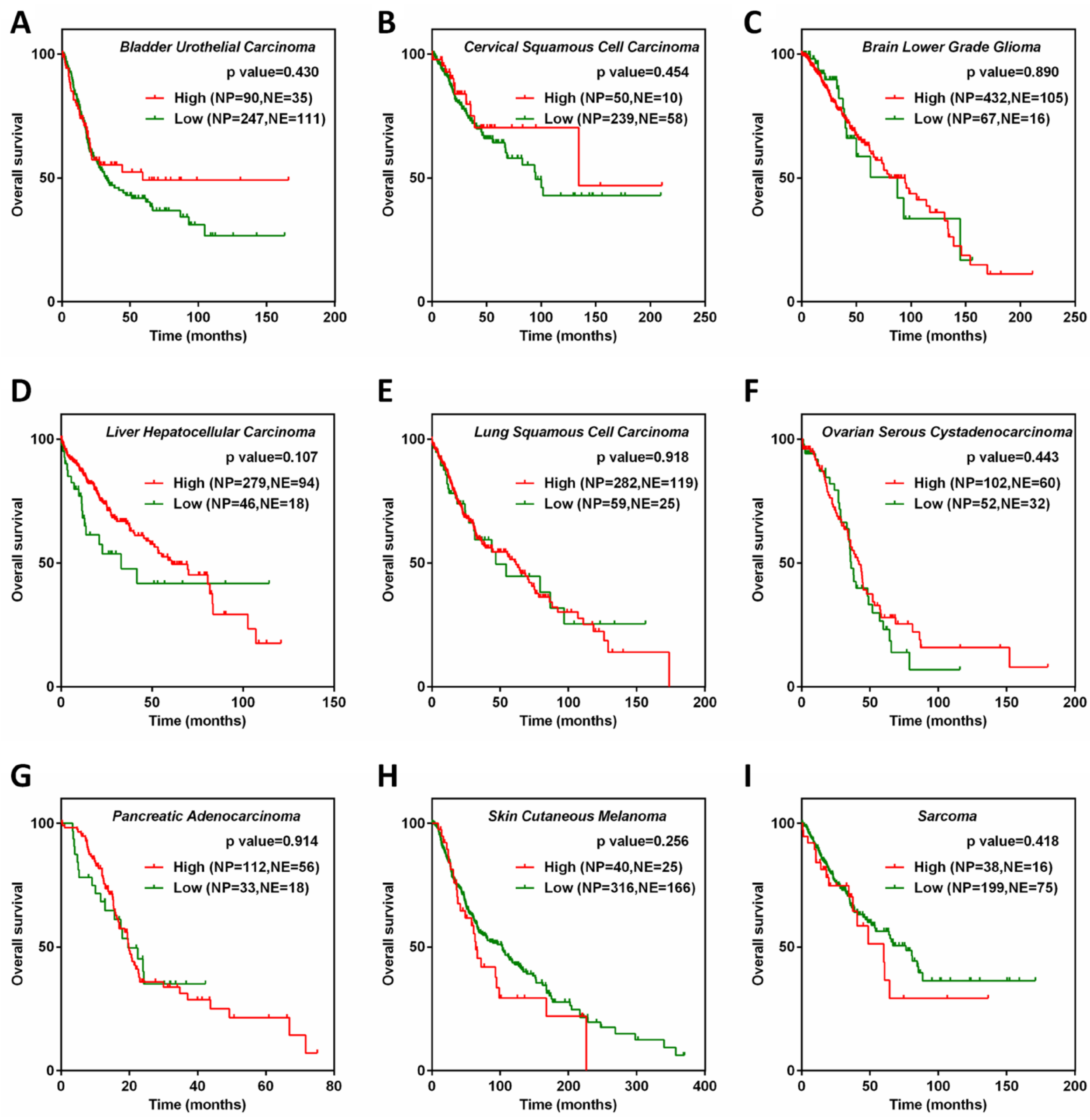

Supplementary Figure 7: Kaplan-Meier curves for GPD1 in bladder urothelial carcinoma (**A**), cervical squamous cell carcinoma (**B**), brain lower grade glioma (**C**), liver hepatocellular carcinoma (**D**), lung squamous cell carcinoma (**E**), ovarian serous cystadenocarcinoma (**F**), pancreatic adenocarcinoma (**G**), skin cutaneous melanoma (**H**), sarcoma (**I**) patients.

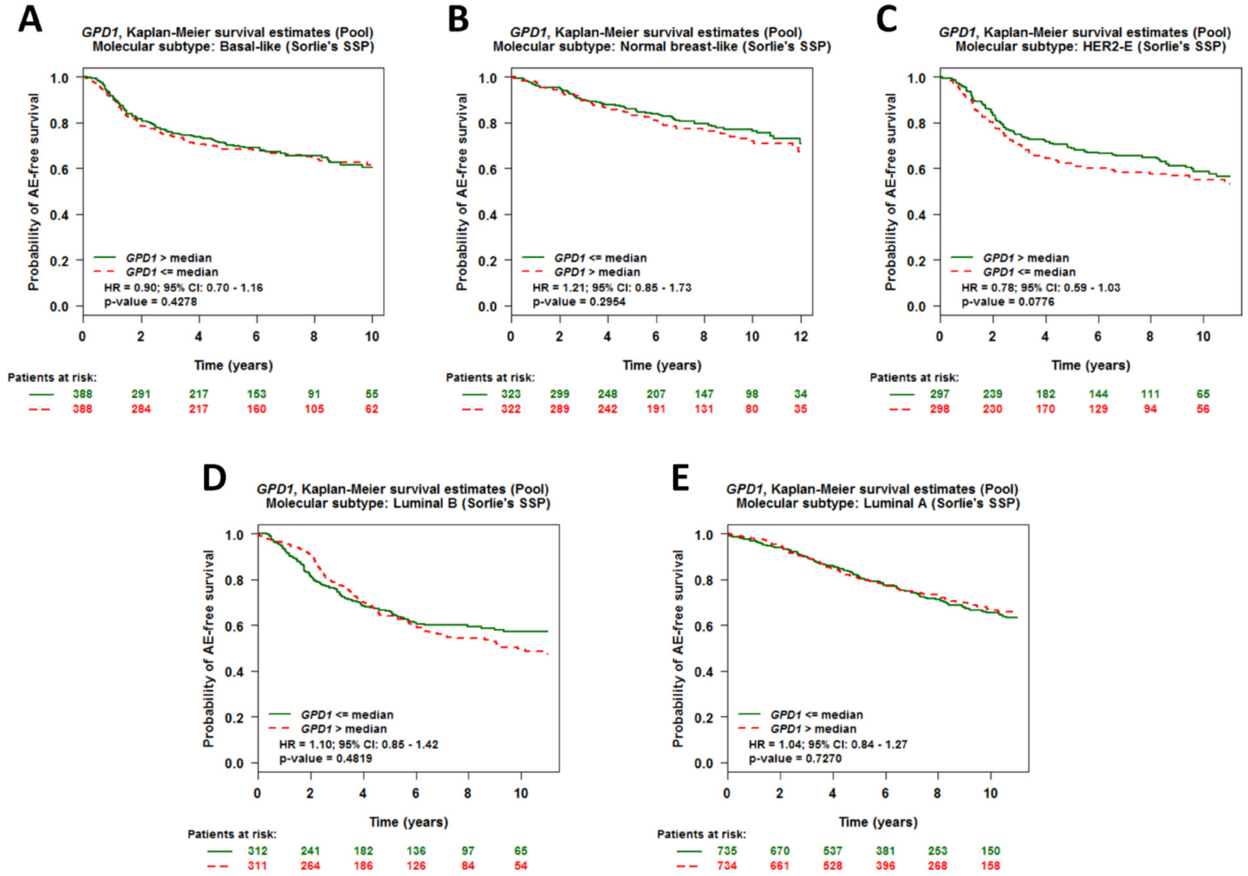

Supplementary Figure 8: Kaplan-Meier curves analysis for GPD1 with patients in basal-like (**A**), normal breast-like (**B**), HER2-E (**C**), luminal B (**D**) and luminal A (**E**) subtype.

Supplementary Table 1: Targeted prognostic analyses for GPD1 with mixed nodal status and mixed ER status patients with metastatic relapse information (metastatic or any relapse, or death) (*n* = 3,875)

***GPD1* univariate Cox analysis (Nm; ERm)**						
**Cohort**	**Reference**	***p*-value**	**HR**	**95% CI**	**No. patients**	**No. MR**
Rosetta2002	Van de Vijver et al., 2002 [1]	0.8022	0.98	0.80–1.19	295	101
PNAS1732912100	Sotiriou et al., 2003 [2]	0.2262	0.7	0.39–1.25	99	30
GSE2603	Minn et al., 2005 [3]	0.6485	0.9	0.57–1.42	82	27
GSE1456	Pawitan et al., 2005 [4]	0.06	0.67	0.44–1.02	159	40
GSE2034	Wang et al., 2005 [5]	0.0876	0.81	0.64–1.03	286	107
E_TABM_158	Chin et al., 2006 [6]	0.4122	1.3	0.69–2.44	112	21
GSE8757	Chin et al., 2007 [7]	0.8965	1.02	0.75–1.38	171	38
GSE7390	Desmedt et al., 2007 [8]	0.1038	0.77	0.56–1.06	198	62
GSE6532	Loi et al., 2007 [9]	0.0627	0.79	0.61–1.01	393	101
GSE5327	Minn et al., 2007 [10]	0.1925	0.54	0.21–1.37	58	11
GSE7378	Zhou et al., 2007 [11]	0.1847	0.47	0.15–1.44	54	9
GSE7849	Anders et al., 2008 [12]	0.2373	0.68	0.36–1.29	75	14
GSE9893	Chanrion et al., 2008 [13]	0.139	0.79	0.57–1.08	155	48
GSE9195	Loi et al., 2008 [14]	0.8537	0.91	0.35–2.37	77	10
GSE11121	Schmidt et al., 2008 [15]	0.9515	1.01	0.74–1.38	200	46
GSE12093	Zhang et al., 2009 [16]	0.1528	0.65	0.36–1.18	136	20
GSE19615	Li et al., 2010 [17]	0.1746	0.52	0.21–1.33	115	14
GSE17907	Sircoulomb et al., 2010 [18]	0.4312	1.38	0.62–3.06	39	17
GSE22219	Buffa et al., 2011 [19]	0.328	0.89	0.71–1.12	216	82
GSE26971	Filipits et al., 2011 [20]	0.8656	1.03	0.77–1.37	258	58
GSE25055	Hatzis et al., 2011 [21]	0.6619	1.06	0.83–1.35	309	65
GSE20685	Kao et al., 2011 [22]	0.5149	0.89	0.63–1.27	296	63
GSE33926	Kuo et al., 2012 [23]	0.0913	1.83	0.91–3.68	51	12
GSE45255	Nagalla et al., 2013 [24]	0.7379	1.09	0.66–1.79	41	14
	Pool	**0.0013**	**0.89**	**0.83–0.96**	**3875**	**1010**

95% CI: 95% confidence interval (values are rounded to 2 decimal places); HR: hazard ratio (values are rounded to 2 decimal places); "Pool" consists in merging all cohorts pooled together with data from all studies previously converted to a common scale with a suitable normalisation (median centred [0] and standard deviation normalized to one).

Supplementary Table 2: Targeted prognostic analyses for GPD1 with mixed nodal status and mixed ER status patients with any event information (metastatic or any relapse, or death) (*n* = 5,488)

GPD1 univariate Cox analysis (Nm; ERm)						
**Cohort**	**Reference**	***p*-value**	**HR**	**95% CI**	**No. patients**	**No. AE**
Rosetta2002	Van de Vijver et al., 2002 [1]	0.851	0.98	0.83–1.17	295	122
PNAS1732912100	Sotiriou et al., 2003 [2]	0.2999	0.79	0.51–1.23	99	53
GSE1378	Ma et al., 2004 [25]	0.9399	0.97	0.50–1.90	59	27
GSE2603	Minn et al., 2005 [3]	0.6485	0.9	0.57–1.42	82	27
GSE1456	Pawitan et al., 2005 [4]	0.2453	0.82	0.59–1.15	159	50
GSE2034	Wang et al., 2005 [5]	0.0876	0.81	0.64–1.03	286	107
GSE3143	Bild et al., 2006 [26]	0.7881	1.04	0.77–1.40	158	50
E_TABM_158	Chin et al., 2006 [6]	0.7865	1.06	0.70–1.61	112	42
GSE4922	Ivshina et al., 2006 [27]	0.11	0.82	0.64–1.05	249	89
GSE8757	Chin et al., 2007 [7]	0.6401	1.06	0.83–1.36	171	56
GSE7390	Desmedt et al., 2007 [8]	0.2622	0.87	0.68–1.11	198	91
GSE6532	Loi et al., 2007 [9]	0.0949	0.84	0.68–1.03	393	139
GSE5327	Minn et al., 2007 [10]	0.1925	0.54	0.21–1.37	58	11
E_UCON_1	Naderi et al., 2007 [28]	0.1945	0.86	0.68–1.08	135	65
GSE7378	Zhou et al., 2007 [11]	0.1847	0.47	0.15–1.44	54	9
GSE7849	Anders et al., 2008 [12]	0.2373	0.68	0.36–1.29	75	14
GSE9893	Chanrion et al., 2008 [13]	0.1876	0.83	0.62–1.10	155	57
GSE9195	Loi et al., 2008 [14]	0.9583	0.98	0.43–2.25	77	13
GSE11121	Schmidt et al., 2008 [15]	0.9515	1.01	0.74–1.38	200	46
GSE10510	Calabrò et al., 2009 [29]	0.0522	0.81	0.65–1.00	134	96
GSE16391	Desmedt et al., 2009 [30]	0.4149	0.87	0.63–1.21	55	55
GSE12093	Zhang et al., 2009 [16]	0.1528	0.65	0.36–1.18	136	20
GSE22133	Jönsson et al., 2010 [31]	0.9997	1	0.84–1.19	339	151
GSE19615	Li et al., 2010 [17]	0.1746	0.52	0.21–1.33	115	14
GSE17907	Sircoulomb et al., 2010 [18]	0.4312	1.38	0.62–3.06	39	17
GSE22219	Buffa et al., 2011 [19]	0.328	0.89	0.71–1.12	216	82
GSE20711	Dedeurwaerder et al., 2011 [32]	0.8946	0.97	0.57–1.63	85	36
GSE26971	Filipits et al., 2011 [20]	0.8656	1.03	0.77–1.37	258	58
GSE25055	Hatzis et al., 2011 [21]	0.6619	1.06	0.83–1.35	309	65
GSE20685	Kao et al., 2011 [22]	0.6754	0.94	0.69–1.27	296	73
GSE21653	Sabatier et al., 2011 [33]	0.9328	0.99	0.74–1.32	252	83
GSE16987	Wang et al., 2011 [34]	0.3093	0.68	0.33–1.42	147	10
GSE33926	Kuo et al., 2012 [23]	0.0913	1.83	0.91–3.68	51	12
GSE45255	Nagalla et al., 2013 [24]	0.7379	1.09	0.66–1.79	41	14
	Pool	0.0005	0.91	0.86–0.96	5488	1854

Supplementary Table 3: Targeted prognostic analyses for GPD1 with mixed nodal status and positive ER status patients with metastatic relapse information (n = 3,917)

Cohort	Reference	*p*-value	HR	95% CI	No. patients	No. AE
Rosetta2002	Van de Vijver et al., 2002 [1]	0.7757	1.03	0.84–1.27	226	82
PNAS1732912100	Sotiriou et al., 2003 [2]	0.5262	0.84	0.49–1.44	65	31
GSE1378	Ma et al., 2004 [25]	0.9399	0.97	0.50–1.90	59	27
GSE2603	Minn et al., 2005 [3]	0.6778	1.15	0.60–2.19	46	13
GSE1456	Pawitan et al., 2005 [4]	0.2336	0.79	0.53–1.17	119	35
GSE2034	Wang et al., 2005 [5]	0.0214	0.72	0.54–0.95	209	80
GSE3143	Bild et al., 2006 [26]	0.8001	1.05	0.73–1.52	110	37
E_TABM_158	Chin et al., 2006 [6]	0.8876	1.05	0.53–2.09	70	26
GSE4922	Ivshina et al., 2006 [27]	0.4024	0.89	0.68–1.16	211	76
GSE8757	Chin et al., 2007 [7]	0.7449	1.05	0.80–1.37	113	36
GSE7390	Desmedt et al., 2007 [8]	0.4048	0.89	0.66–1.18	134	59
GSE6532	Loi et al., 2007 [9]	0.1373	0.84	0.66–1.06	348	117
E_UCON_1	Naderi et al., 2007 [28]	0.1268	0.8	0.61–1.06	93	44
GSE7378	Zhou et al., 2007 [11]	0.1847	0.47	0.15–1.44	54	9
GSE7849	Anders et al., 2008 [12]	0.1394	0.46	0.16–1.29	46	6
GSE9893	Chanrion et al., 2008 [13]	0.0826	0.75	0.54–1.04	135	50
GSE9195	Loi et al., 2008 [14]	0.9583	0.98	0.43–2.25	77	13
GSE11121	Schmidt et al., 2008 [15]	0.9644	0.99	0.70–1.41	154	34
GSE10510	Calabrò et al., 2009 [29]	0.32	0.84	0.61–1.18	81	57
GSE16391	Desmedt et al., 2009 [30]	0.4149	0.87	0.63–1.21	55	55
GSE12093	Zhang et al., 2009 [16]	0.1528	0.65	0.36–1.18	136	20
GSE22133	Jönsson et al., 2010 [31]	0.6465	1.05	0.86–1.28	219	92
GSE19615	Li et al., 2010 [17]	0.5005	0.57	0.11–2.93	70	4
GSE17907	Sircoulomb et al., 2010 [18]	0.6016	0.6	0.09–4.12	14	4
GSE22219	Buffa et al., 2011 [19]	0.7346	0.95	0.71–1.27	134	49
GSE20711	Dedeurwaerder et al., 2011 [32]	0.7005	0.86	0.38–1.90	41	13
GSE26971	Filipits et al., 2011 [20]	0.8656	1.03	0.77–1.37	258	58
GSE25055	Hatzis et al., 2011 [21]	0.7742	1.06	0.73–1.53	173	23
GSE20685	Kao et al., 2011 [22]	0.5688	0.89	0.59–1.33	188	40
GSE21653	Sabatier et al., 2011 [33]	0.4299	0.85	0.56–1.28	140	43
GSE16987	Wang et al., 2011 [34]	0.7968	1.11	0.51–2.39	112	5
GSE45255	Nagalla et al., 2013 [24]	0.6414	1.13	0.67–1.90	27	10
	Pool	0.0027	0.91	0.85–0.97	3917	1248

Supplementary Table 4: Targeted prognostic analyses for GPD1 with mixed nodal status andmixed ER status patients with any event information (metastatic or any relapse, or death) (*n* = 5,488)

GPD1 univariate Cox analysis (Nm; ERm)							
**Cohort**	**Reference**	***p*-value**	**HR**	**95% CI**	**No. patients**	**No. AE**
Rosetta2002	Van de Vijver et al., 2002 [1]	0.851	0.98	0.83–1.17	295	122
PNAS1732912100	Sotiriou et al., 2003 [2]	0.2999	0.79	0.51–1.23	99	53
GSE1378	Ma et al., 2004 [25]	0.9399	0.97	0.50–1.90	59	27
GSE2603	Minn et al., 2005 [3]	0.6485	0.9	0.57–1.42	82	27
GSE1456	Pawitan et al., 2005 [4]	0.2453	0.82	0.59–1.15	159	50
GSE2034	Wang et al., 2005 [5]	0.0876	0.81	0.64–1.03	286	107
GSE3143	Bild et al., 2006 [26]	0.7881	1.04	0.77–1.40	158	50
E_TABM_158	Chin et al., 2006 [6]	0.7865	1.06	0.70–1.61	112	42
GSE4922	Ivshina et al., 2006 [27]	0.11	0.82	0.64–1.05	249	89
GSE8757	Chin et al., 2007 [7]	0.6401	1.06	0.83–1.36	171	56
GSE7390	Desmedt et al., 2007 [8]	0.2622	0.87	0.68–1.11	198	91
GSE6532	Loi et al., 2007 [9]	0.0949	0.84	0.68–1.03	393	139
GSE5327	Minn et al., 2007 [10]	0.1925	0.54	0.21–1.37	58	11
E_UCON_1	Naderi et al., 2007 [28]	0.1945	0.86	0.68–1.08	135	65
GSE7378	Zhou et al., 2007 [11]	0.1847	0.47	0.15–1.44	54	9
GSE7849	Anders et al., 2008 [12]	0.2373	0.68	0.36–1.29	75	14
GSE9893	Chanrion et al., 2008 [13]	0.1876	0.83	0.62–1.10	155	57
GSE9195	Loi et al., 2008 [14]	0.9583	0.98	0.43–2.25	77	13
GSE11121	Schmidt et al., 2008 [15]	0.9515	1.01	0.74–1.38	200	46
GSE10510	Calabrò et al., 2009 [29]	0.0522	0.81	0.65–1.00	134	96
GSE16391	Desmedt et al., 2009 [30]	0.4149	0.87	0.63–1.21	55	55
GSE12093	Zhang et al., 2009 [16]	0.1528	0.65	0.36–1.18	136	20
GSE22133	Jönsson et al., 2010 [31]	0.9997	1	0.84–1.19	339	151
GSE19615	Li et al., 2010 [17]	0.1746	0.52	0.21–1.33	115	14
GSE17907	Sircoulomb et al., 2010 [18]	0.4312	1.38	0.62–3.06	39	17
GSE22219	Buffa et al., 2011 [19]	0.328	0.89	0.71–1.12	216	82
GSE20711	Dedeurwaerder et al., 2011 [32]	0.8946	0.97	0.57–1.63	85	36
GSE26971	Filipits et al., 2011 [20]	0.8656	1.03	0.77–1.37	258	58
GSE25055	Hatzis et al., 2011 [21]	0.6619	1.06	0.83–1.35	309	65
GSE20685	Kao et al., 2011 [22]	0.6754	0.94	0.69–1.27	296	73
GSE21653	Sabatier et al., 2011 [33]	0.9328	0.99	0.74–1.32	252	83
GSE16987	Wang et al., 2011 [34]	0.3093	0.68	0.33–1.42	147	10
GSE33926	Kuo et al., 2012 [23]	0.0913	1.83	0.91–3.68	51	12
GSE45255	Nagalla et al., 2013 [24]	0.7379	1.09	0.66–1.79	41	14
	Pool	0.0005	0.91	0.86–0.96	5488	1854

Supplementary Table 5: Targeted prognostic analyses for GPD1 with nagetive nodal status andmixed ER status patients with any event information (metastatic or any relapse, or death) (*n* = 2,456)

Cohort	Reference	*p*-value	HR	95% CI	No. patients	No. AE
Rosetta2002	Van de Vijver et al., 2002 [1]	0.6858	1.06	0.81–1.37	151	65
PNAS1732912100	Sotiriou et al., 2003 [2]	0.0839	0.51	0.24–1.09	46	18
GSE1378	Ma et al., 2004 [25]	0.2194	0.54	0.20–1.45	27	12
GSE2603	Minn et al., 2005 [3]	0.4996	1.36	0.56–3.32	28	9
GSE2034	Wang et al., 2005 [5]	0.0876	0.81	0.64–1.03	286	107
E_TABM_158	Chin et al., 2006 [6]	0.954	1.02	0.57–1.82	49	16
GSE4922	Ivshina et al., 2006 [27]	0.4971	0.9	0.66–1.23	159	49
GSE7390	Desmedt et al., 2007 [8]	0.2622	0.87	0.68–1.11	198	91
GSE6532	Loi et al., 2007 [9]	0.2087	0.85	0.66–1.10	241	84
E_UCON_1	Naderi et al., 2007 [28]	0.4542	0.89	0.65–1.21	86	33
GSE7378	Zhou et al., 2007 [11]	0.1847	0.47	0.15–1.44	54	9
GSE7849	Anders et al., 2008 [12]	0.3765	0.68	0.29–1.59	46	6
GSE9893	Chanrion et al., 2008 [13]	0.9808	0.99	0.56–1.75	78	14
GSE9195	Loi et al., 2008 [14]	0.3131	0.35	0.05–2.66	41	3
GSE11121	Schmidt et al., 2008 [15]	0.9515	1.01	0.74–1.38	200	46
GSE10510	Calabrò et al., 2009 [29]	0.0672	0.68	0.45–1.03	35	24
GSE16391	Desmedt et al., 2009 [30]	0.7826	1.09	0.58–2.06	22	22
GSE12093	Zhang et al., 2009 [16]	0.1528	0.65	0.36–1.18	136	20
GSE19615	Li et al., 2010 [17]	0.5462	0.67	0.18–2.48	64	5
GSE17907	Sircoulomb et al., 2010 [18]	0.287	2.71	0.43–17.00	11	4
GSE22219	Buffa et al., 2011 [19]	0.209	0.79	0.54–1.14	125	35
GSE20711	Dedeurwaerder et al., 2011 [32]	0.688	0.75	0.18–3.12	29	7
GSE25055	Hatzis et al., 2011 [21]	0.5425	1.19	0.68–2.07	87	6
GSE21653	Sabatier et al., 2011 [33]	0.3176	0.76	0.44–1.30	116	32
GSE16987	Wang et al., 2011 [34]	0.0619	0.17	0.03–1.09	70	4
GSE33926	Kuo et al., 2012 [23]	0.1528	2.77	0.69–11.16	30	4
GSE45255	Nagalla et al., 2013 [24]	0.7379	1.09	0.66–1.79	41	14
	Pool	0.0013	0.87	0.80–0.95	2456	739

Supplementary Table 6: Molecular subtype prognostic analyses for GPD1 with patients having mixed event information (metastatic or any relapse, or death) (n = 5,488)

GPD1 univariate Cox analysis by SSP molecular subtype						
**Molecular subtype**	**Analysis results**	**Single Sample Predictors**			**RSSPC**	**RMSPC**
		**Sorlie's**	**Hu's**	**PAM50**		
Basal-like	p-value	0.3761	0.5403	0.4801	0.455	0.8465
	HR	0.93	0.96	1.05	0.93	0.98
	95% CI	0.78–1.10	0.84–1.10	0.92–1.18	0.78–1.12	0.78–1.22
	No. patients	776	1243	1120	687	568
	No. events	253	448	393	222	189
HER2-E	p-value	0.0051	0.0386	0.1069	0.1092	0.5665
	HR	0.75	0.79	0.88	0.72	0.87
	95% CI	0.61–0.92	0.64–0.99	0.75–1.03	0.48–1.08	0.54–1.40
	No. patients	595	485	806	184	119
	No. events	232	204	332	76	47
Luminal A	p-value	0.5238	0.8571	0.8214	0.6506	0.2442
	HR	0.96	1.01	0.99	0.95	1.23
	95% CI	0.85–1.09	0.88–1.16	0.88–1.10	0.76–1.19	0.87–1.73
	No. patients	1469	1310	1537	739	316
	No. events	388	285	372	141	58
Luminal B	p-value	0.9927	0.9856	0.4408	0.9317	0.6019
	HR	1.00	1.00	0.95	0.98	0.88
	95% CI	0.84–1.19	0.88–1.13	0.84–1.08	0.69–1.40	0.53–1.44
	No. patients	623	970	1050	186	77
	No. events	253	430	422	96	43
Normal breast-like	p-value	0.2728	0.4635	0.2002	0.2755	
	HR	1.09	1.05	1.09	1.12	
	95% CI	0.94–1.26	0.92–1.20	0.95–1.25	0.92–1.36	
	No. patients	645	783	715	325	
	No. events	151	203	204	78	
Unclassified	No. patients	1380	697	260		

SSP: Single Sample Predictor (Sorlie's, Hu's and PAM50); SCM: Subtype Clustering Model (SCMOD1 [35], SCMOD2 [36] and SCMGENE [37]); MSP: Molecular Subtype Predictor (SSPs + SCMs); RMSPC: Robust Molecular Subtype Predictors Classification based on patients classified in the same subtype with the six MSPs; RSSPC: Robust SSP Classification based on patients classified in the same subtype with the three SSPs.

Supplementary Table 7: Correlation of GPD1 expression with copy number changes in different cancer types

Cancer type	Shallow deletiion	Diploid	Gain	p value (shallow deletion vs diploid)	p value (gain vs diploid)
Adrenocortical Carcinoma	1	18	58	ND	0.1862
Bladder Urothelial Carcinoma	65	248	91	> 0.99	0.0188
Colorectal Adenocarcinoma	38	264	74	0.0559	0.1236
Breast Invasive Carcinoma	162	699	214	< 0.0001	0.0002
Cervical Squamous Cell Carcinoma	16	224	52	0.1336	0.0312
Brain Lower Grade Glioma	60	442	11	0.0012	0.7799
Glioblastoma Multiforme	14	116	17	0.1416	0.7721
Esophageal Carcinoma	39	110	34	0.4788	0.4323
Stomach Adenocarcinoma	3	26	7	ND	ND
Uveal Melanoma	2	75	3	ND	ND
Head and Neck Squamous Cell Carcinoma	52	378	84	0.0527	0.1688
Kidney Renal Clear Cell Carcinoma	5	401	119	ND	0.1333
Liver Hepatocellular Carcinoma	37	280	47	0.0519	0.0008
Lung Adenocarcinoma	95	275	142	0.2487	0.0023
Lung Squamous Cell Carcinoma	59	288	151	0.0034	0.5544
Ovarian Serous Cystadenocarcinoma	53	144	104	0.0195	0.2711
Pancreatic Adenocarcinoma	33	120	24	< 0.0001	0.6495
Mesothelioma	5	64	17	ND	0.4102
Prostate Adenocarcinoma	17	304	11	0.3888	0.9893
Skin Cutaneous Melanoma	77	249	40	0.4238	0.0001
Sarcoma	68	132	38	0.0458	< 0.0001

ND: not done, because of no cases or a small number of cases (< 10).

REFERENCES

1. van de Vijver MJ, He YD, van't Veer LJ, Dai H, Hart AA, Voskuil DW, Schreiber GJ, Peterse JL, Roberts C, Marton MJ, Parrish M, Atsma D, Witteveen A, et al. A gene-expression signature as a predictor of survival in breast cancer. N Engl J Med. 2002; 347:1999–2009.
2. Sotiriou C, Neo SY, McShane LM, Korn EL, Long PM, Jazaeri A, Martiat P, Fox SB, Harris AL and Liu ET. Breast cancer classification and prognosis based on gene expression profiles from a population-based study. Proc Natl Acad Sci U S A. 2003; 100:10393–10398.
3. Minn AJ, Gupta GP, Siegel PM, Bos PD, Shu W, Giri DD, Viale A, Olshen AB, Gerald WL and Massague J. Genes that mediate breast cancer metastasis to lung. Nature. 2005; 436:518–524.
4. Pawitan Y, Bjohle J, Amler L, Borg AL, Egyhazi S, Hall P, Han X, Holmberg L, Huang F, Klaar S, Liu ET, Miller L, Nordgren H, et al. Gene expression profiling spares early breast cancer patients from adjuvant therapy: derived and validated in two population-based cohorts. Breast Cancer Res. 2005; 7:R953-964.
5. Wang Y, Klijn JG, Zhang Y, Sieuwerts AM, Look MP, Yang F, Talantov D, Timmermans M, Meijer-van Gelder ME, Yu J, Jatkoe T, Berns EM, Atkins D and Foekens JA. Gene-expression profiles to predict distant metastasis of lymph-node-negative primary breast cancer. Lancet. 2005; 365:671–679.
6. Chin K, DeVries S, Fridlyand J, Spellman PT, Roydasgupta R, Kuo WL, Lapuk A, Neve RM, Qian Z, Ryder T, Chen F, Feiler H, Tokuyasu T, et al. Genomic and transcriptional aberrations linked to breast cancer pathophysiologies. Cancer Cell. 2006; 10:529–541.
7. Chin SF, Teschendorff AE, Marioni JC, Wang Y, Barbosa-Morais NL, Thorne NP, Costa JL, Pinder SE, van de Wiel MA, Green AR, Ellis IO, Porter PL, Tavare S, et al. High-resolution aCGH and expression profiling identifies a novel genomic subtype of ER negative breast cancer. Genome Biol. 2007; 8:R215.
8. Desmedt C, Piette F, Loi S, Wang Y, Lallemand F, Haibe-Kains B, Viale G, Delorenzi M, Zhang Y, d'Assignies MS, Bergh J, Lidereau R, Ellis P, et al. Strong time dependence of the 76-gene prognostic signature for node-negative breast cancer patients in the TRANSBIG multicenter independent validation series. Clin Cancer Res. 2007; 13:3207–3214.
9. Loi S, Haibe-Kains B, Desmedt C, Lallemand F, Tutt AM, Gillet C, Ellis P, Harris A, Bergh J, Foekens JA, Klijn JG, Larsimont D, Buyse M, et al. Definition of clinically distinct molecular subtypes in estrogen receptor-positive breast carcinomas through genomic grade. J Clin Oncol. 2007; 25:1239–1246.
10. Minn AJ, Gupta GP, Padua D, Bos P, Nguyen DX, Nuyten D, Kreike B, Zhang Y, Wang Y, Ishwaran H, Foekens JA, van de Vijver M and Massague J. Lung metastasis genes couple breast tumor size and metastatic spread. Proc Natl Acad Sci U S A. 2007; 104:6740–6745.
11. Zhou Y, Yau C, Gray JW, Chew K, Dairkee SH, Moore DH, Eppenberger U, Eppenberger-Castori S and Benz CC. Enhanced NF kappa B and AP-1 transcriptional activity associated with antiestrogen resistant breast cancer. BMC Cancer. 2007; 7:59.
12. Anders CK, Acharya CR, Hsu DS, Broadwater G, Garman K, Foekens JA, Zhang Y, Wang Y, Marcom K, Marks JR, Mukherjee S, Nevins JR, Blackwell KL and Potti A. Age-specific differences in oncogenic pathway deregulation seen in human breast tumors. PLoS One. 2008; 3:e1373.
13. Chanrion M, Negre V, Fontaine H, Salvetat N, Bibeau F, Mac Grogan G, Mauriac L, Katsaros D, Molina F, Theillet C and Darbon JM. A gene expression signature that can predict the recurrence of tamoxifen-treated primary breast cancer. Clin Cancer Res. 2008; 14:1744–1752.
14. Loi S, Haibe-Kains B, Desmedt C, Wirapati P, Lallemand F, Tutt AM, Gillet C, Ellis P, Ryder K, Reid JF, Daidone MG, Pierotti MA, Berns EM, et al. Predicting prognosis using molecular profiling in estrogen receptor-positive breast cancer treated with tamoxifen. BMC Genomics. 2008; 9:239.
15. Schmidt M, Bohm D, von Torne C, Steiner E, Puhl A, Pilch H, Lehr HA, Hengstler JG, Kolbl H and Gehrmann M. The humoral immune system has a key prognostic impact in node-negative breast cancer. Cancer Res. 2008; 68:5405–5413.
16. Zhang Y, Sieuwerts AM, McGreevy M, Casey G, Cufer T, Paradiso A, Harbeck N, Span PN, Hicks DG, Crowe J, Tubbs RR, Budd GT, Lyons J, et al. The 76-gene signature defines high-risk patients that benefit from adjuvant tamoxifen therapy. Breast Cancer Res Treat. 2009; 116:303–309.
17. Li Y, Zou L, Li Q, Haibe-Kains B, Tian R, Li Y, Desmedt C, Sotiriou C, Szallasi Z, Iglehart JD, Richardson AL and Wang ZC. Amplification of LAPTM4B and YWHAZ contributes to chemotherapy resistance and recurrence of breast cancer. Nat Med. 2010; 16:214–218.
18. Sircoulomb F, Bekhouche I, Finetti P, Adelaide J, Ben Hamida A, Bonansea J, Raynaud S, Innocenti C, Charafe-Jauffret E, Tarpin C, Ben Ayed F, Viens P, Jacquemier J, et al. Genome profiling of ERBB2-amplified breast cancers. BMC Cancer. 2010; 10:539.
19. Buffa FM, Camps C, Winchester L, Snell CE, Gee HE, Sheldon H, Taylor M, Harris AL and Ragoussis J. microRNA-associated progression pathways and potential therapeutic targets identified by integrated mRNA and microRNA expression profiling in breast cancer. Cancer Res. 2011; 71:5635–5645.
20. Filipits M, Rudas M, Jakesz R, Dubsky P, Fitzal F, Singer CF, Dietze O, Greil R, Jelen A, Sevelda P, Freibauer C, Muller V, Janicke F, et al. A new molecular predictor of distant recurrence in ER-positive, HER2-negative breast cancer adds independent information to conventional clinical risk factors. Clin Cancer Res. 2011; 17:6012–6020.
21. Hatzis C, Pusztai L, Valero V, Booser DJ, Esserman L, Lluch A, Vidaurre T, Holmes F, Souchon E, Wang H, Martin M, Cotrina J, Gomez H, et al. A genomic predictor of response and survival following taxane-anthracycline chemotherapy for invasive breast cancer. Jama. 2011; 305:1873–1881.

22. Kao KJ, Chang KM, Hsu HC and Huang AT. Correlation of microarray-based breast cancer molecular subtypes and clinical outcomes: implications for treatment optimization. BMC Cancer. 2011; 11:143.
23. Kuo WH, Chang YY, Lai LC, Tsai MH, Hsiao CK, Chang KJ and Chuang EY. Molecular characteristics and metastasis predictor genes of triple-negative breast cancer: a clinical study of triple-negative breast carcinomas. PLoS One. 2012; 7:e45831.
24. Nagalla S, Chou JW, Willingham MC, Ruiz J, Vaughn JP, Dubey P, Lash TL, Hamilton-Dutoit SJ, Bergh J, Sotiriou C, Black MA and Miller LD. Interactions between immunity, proliferation and molecular subtype in breast cancer prognosis. Genome Biol. 2013; 14:R34.
25. Ma XJ, Wang Z, Ryan PD, Isakoff SJ, Barmettler A, Fuller A, Muir B, Mohapatra G, Salunga R, Tuggle JT, Tran Y, Tran D, Tassin A, et al. A two-gene expression ratio predicts clinical outcome in breast cancer patients treated with tamoxifen. Cancer Cell. 2004; 5:607–616.
26. Bild AH, Yao G, Chang JT, Wang Q, Potti A, Chasse D, Joshi MB, Harpole D, Lancaster JM, Berchuck A, Olson JA, Jr., Marks JR, et al. Oncogenic pathway signatures in human cancers as a guide to targeted therapies. Nature. 2006; 439:353–357.
27. Ivshina AV, George J, Senko O, Mow B, Putti TC, Smeds J, Lindahl T, Pawitan Y, Hall P, Nordgren H, Wong JE, Liu ET, Bergh J, et al. Genetic reclassification of histologic grade delineates new clinical subtypes of breast cancer. Cancer Res. 2006; 66:10292–10301.
28. Naderi A, Teschendorff AE, Barbosa-Morais NL, Pinder SE, Green AR, Powe DG, Robertson JF, Aparicio S, Ellis IO, Brenton JD and Caldas C. A gene-expression signature to predict survival in breast cancer across independent data sets. Oncogene. 2007; 26:1507–1516.
29. Calabro A, Beissbarth T, Kuner R, Stojanov M, Benner A, Asslaber M, Ploner F, Zatloukal K, Samonigg H, Poustka A and Sultmann H. Effects of infiltrating lymphocytes and estrogen receptor on gene expression and prognosis in breast cancer. Breast Cancer Res Treat. 2009; 116:69–77.
30. Desmedt C, Giobbie-Hurder A, Neven P, Paridaens R, Christiaens MR, Smeets A, Lallemand F, Haibe-Kains B, Viale G, Gelber RD, Piccart M and Sotiriou C. The Gene expression Grade Index: a potential predictor of relapse for endocrine-treated breast cancer patients in the BIG 1-98 trial. BMC Med Genomics. 2009; 2:40.
31. Jonsson G, Staaf J, Vallon-Christersson J, Ringner M, Holm K, Hegardt C, Gunnarsson H, Fagerholm R, Strand C, Agnarsson BA, Kilpivaara O, Luts L, Heikkila P, et al. Genomic subtypes of breast cancer identified by array-comparative genomic hybridization display distinct molecular and clinical characteristics. Breast Cancer Res. 2010; 12:R42.
32. Dedeurwaerder S, Desmedt C, Calonne E, Singhal SK, Haibe-Kains B, Defrance M, Michiels S, Volkmar M, Deplus R, Luciani J, Lallemand F, Larsimont D, Toussaint J, et al. DNA methylation profiling reveals a predominant immune component in breast cancers. EMBO Mol Med. 2011; 3:726–741.
33. Sabatier R, Finetti P, Cervera N, Lambaudie E, Esterni B, Mamessier E, Tallet A, Chabannon C, Extra JM, Jacquemier J, Viens P, Birnbaum D and Bertucci F. A gene expression signature identifies two prognostic subgroups of basal breast cancer. Breast Cancer Res Treat. 2011; 126:407–420.
34. Wang DY, Done SJ, McCready DR, Boerner S, Kulkarni S and Leong WL. A new gene expression signature, the ClinicoMolecular Triad Classification, may improve prediction and prognostication of breast cancer at the time of diagnosis. Breast Cancer Res. 2011; 13:R92.
35. Desmedt C, Haibe-Kains B, Wirapati P, Buyse M, Larsimont D, Bontempi G, Delorenzi M, Piccart M and Sotiriou C. Biological processes associated with breast cancer clinical outcome depend on the molecular subtypes. Clin Cancer Res. 2008; 14:5158–5165.
36. Wirapati P, Sotiriou C, Kunkel S, Farmer P, Pradervand S, Haibe-Kains B, Desmedt C, Ignatiadis M, Sengstag T, Schutz F, Goldstein DR, Piccart M and Delorenzi M. Meta-analysis of gene expression profiles in breast cancer: toward a unified understanding of breast cancer subtyping and prognosis signatures. Breast Cancer Res. 2008; 10:R65.
37. Haibe-Kains B, Desmedt C, Rothe F, Piccart M, Sotiriou C and Bontempi G. A fuzzy gene expression-based computational approach improves breast cancer prognostication. Genome Biol. 2010; 11:R18.
